# Host genetic background is a barrier to broadly effective vaccine–mediated protection against tuberculosis

**DOI:** 10.1172/JCI167762

**Published:** 2023-07-03

**Authors:** Rocky Lai, Diana N. Gong, Travis Williams, Abiola F. Ogunsola, Kelly Cavallo, Cecilia S. Lindestam Arlehamn, Sarah Acolatse, Gillian L. Beamer, Martin T. Ferris, Christopher M. Sassetti, Douglas A. Lauffenburger, Samuel M. Behar

**Affiliations:** 1 Department of Microbiology and Physiological Systems, University of Massachusetts Medical School, Worcester, Massachusetts, USA.; 2 Department of Biological Engineering, Massachusetts Institute of Technology, Cambridge, Massachusetts, USA.; 3 Center for infectious disease and vaccine research, La Jolla Institute for Immunology, La Jolla, California, USA.; 4 Texas Biomedical Research Institute, San Antonio, Texas, USA.; 5 Department of Genetics, University of North Carolina at Chapel Hill, Chapel Hill, North Carolina, USA.

**Keywords:** Infectious disease, Vaccines, Genetic variation, Mouse models, Tuberculosis

## Abstract

Heterogeneity in human immune responses is difficult to model in standard laboratory mice. To understand how host variation affects Bacillus Calmette Guerin–induced (BCG-induced) immunity against *Mycobacterium tuberculosis*, we studied 24 unique collaborative cross (CC) mouse strains, which differ primarily in the genes and alleles they inherit from founder strains. The CC strains were vaccinated with or without BCG and challenged with aerosolized *M*. *tuberculosis*. Since BCG protects only half of the CC strains tested, we concluded that host genetics has a major influence on BCG-induced immunity against *M*. *tuberculosis* infection, making it an important barrier to vaccine-mediated protection. Importantly, BCG efficacy is dissociable from inherent susceptibility to tuberculosis (TB). T cell immunity was extensively characterized to identify components associated with protection that were stimulated by BCG and recalled after *M*. *tuberculosis* infection. Although considerable diversity is observed, BCG has little impact on the composition of T cells in the lung after infection. Instead, variability is largely shaped by host genetics. BCG-elicited protection against TB correlated with changes in immune function. Thus, CC mice can be used to define correlates of protection and to identify vaccine strategies that protect a larger fraction of genetically diverse individuals instead of optimizing protection for a single genotype.

## Graphical abstract

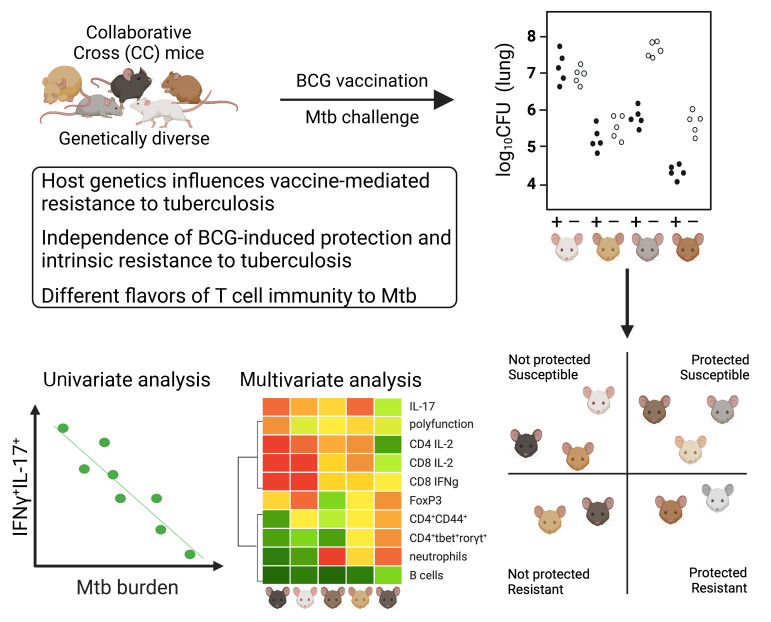

## Introduction

The introduction of Bacillus Calmette Guerin (BCG) as a vaccine contributed significantly to tuberculosis (TB) control, which has threatened global health for millennia. As the only vaccine approved for preventing TB, 4 billion doses of BCG have been administered in 150 countries (1). BCG prevents extrapulmonary TB in infants, but it is less effective in averting pulmonary disease in adolescents and adults (2–5). BCG’s variable efficacy in different geographical regions complicates its use (6). While the development of more effective TB vaccines is an important unmet medical need, our lack of mechanistic insights into how protective immunity against TB is mediated impedes our progress toward this goal. The identification of correlates of protective immunity to TB would accelerate this process by facilitating more rational vaccine design.

Why is identifying immune correlates of protection following vaccination difficult? TB is primarily a human disease that is transmitted person-to-person by aerosolized *Mycobacterium tuberculosis* (Mtb). No animal model recapitulates all the features of human TB, although nonhuman primates come close (7, 8). While imperfect, the mouse model facilitated mechanistic studies that defined many of the critical immunological pathways required for host resistance, permitted in vivo antibiotic testing, and accelerated preclinical vaccine development. As all individuals within an inbred mouse strain are genetically identical, a strength of the mouse model is its “infinite” reproducibility. For experimental immunology, C57BL/6 mice are the preferred inbred strain, as most genetically modified mice are produced using this genetic background. It is also the most frequently used mouse strain for TB research. However, several features of C57BL/6 mice suggest it is an outlier strain for TB research. It expresses a single class II MHC molecule (i.e., I-A^b^) and is missing a class I MHC gene. Importantly, it is among the most resistant of all inbred strains to Mtb infection (9), which makes it difficult to identify conditions that increase its resistance to disease. Investigators have addressed these limitations by using F1 hybrids with susceptible mice to increase MHC and genetic diversity and to identify strategies that increase host resistance (10–13). However, genetic diversity is limited even among classical inbred strains (14). This is a problem that affects many areas of biomedical research.

The mouse-genetics community responded to this problem by developing a resource that captures the natural genetic diversity of *Mus musculus*. Starting with 8 founder strains, which include representatives from all 3 *Mus musculus* subspecies, an 8-way funnel breeding scheme was used to create progeny with a random assortment of the founder genomes (15). The descendants of this effort became the collaborative cross (CC) strains. Each CC strain has a genome that is a unique genetic combination (i.e., genotype) of the 8 founders. As each CC strain is inbred, they are a renewable resource, and unlimited experiments can be performed with the same genotype. Their population structure is well suited to study the impact of genetic variation of host traits. These attributes make the CC ideal for identifying experimental correlates and defining mechanisms. This approach has proven useful in murine models of infection for *Pseudomonas aeruginosa*, Zika virus, and Lymphocytic Choriomeningitis Virus (LCMV) (16–18).

We previously reported that the CC strains have large variations in primary susceptibility to Mtb (19, 20). There is also variation in protection conferred by BCG against Mtb in the CC founder strains, suggesting that the CC strains would be an excellent model to understand how host genetics affects vaccine-induced protection (19). To understand how host variation affects BCG-induced immunity against Mtb and to identify immune correlates of protection, 24 CC mouse strains were vaccinated with BCG or were left unvaccinated as a control, challenged with aerosolized Mtb, and ranked by how well BCG protected these mice against Mtb. Importantly, the host’s genetic background had a major effect on vaccine-induced protection. We hypothesized that CC strains protected by BCG would share common immunological features. However, many of the protected CC strains had unique flavors of immunity, indicating multiple paths to protection. We propose that CC mice can be used as a preclinical model to facilitate the development of vaccines that protect a diverse range of genotypes against Mtb infection.

## Results

### BCG vaccination protects only a subset of CC strains against TB.

**Figure 1:**
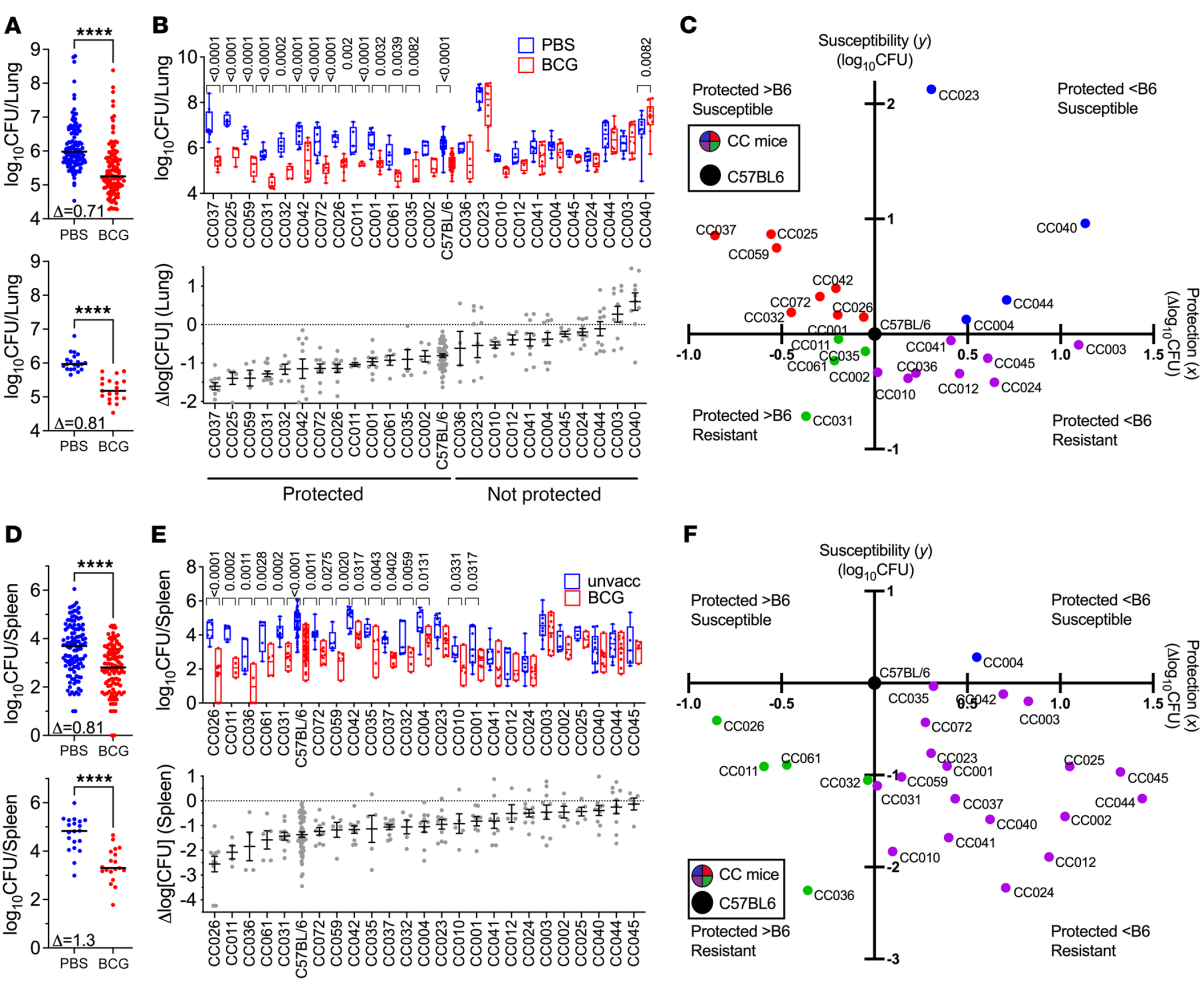
BCG-induced protection in 24 CC strains and C57BL/6 mice challenged with Mtb. (**A**) Lung and (**D**) spleen CFU of 24 CC mice (*n* = 120/group; top) or C57BL/6 mice (*n* = 20/group; bottom) 4 weeks after infection. Each point represents an individual mouse and the line is the median. Δ, Δlog_10_CFU. Student’s *t* test; ****P < 0.0001. CFU in (**B**) lungs and (**E**) spleens from BCG versus unvaccinated strains (*n* = 5/group) after Mtb challenge, represented as box-and-whisker plots, with bounds from 25th to 75th percentile, median line and whiskers ranging from minimum to maximum. The Benjamini-Hochberg procedure was used to determine the FDR (above bars). The Δlog_10_CFU was calculated for each strain (bottom). Correlation in the (**C**) lung and (**F**) spleen between susceptibility (y-axis, CFU in unvaccinated group) and protection conferred by BCG (x-axis, Δlog_10_CFU). The data are relative to the C57BL/6 reference strain, which centers the graph on C57BL/6 mice. The CC strains are divided into 4 quadrants based on their relative susceptibility and protection.

To establish how the host genetic background affects vaccine-mediated protection against Mtb, 24 different CC strains were vaccinated s.c. with BCG or left unvaccinated and rested for 12 weeks. Mice were then infected with low dose aerosolized H37Rv expressing YFP (H37Rv:YFP) (21). Four weeks after infection, lung and spleen CFU were determined. The aggregated lung CFU from the unvaccinated and BCG-vaccinated CC strains (Figure 1A, top) and C57BL/6 mice (Figure 1A, bottom) showed that as a population, CC mice were protected by BCG similarly to C57BL/6 mice, although the variance among the CC strains was greater. When the data are segregated by individual CC strains, one can observe that the population-wide variance reflected strain-specific differences in protection conferred by BCG vaccination (Figure 1B, top). Each CC strain was classified as BCG-protected if the difference (Δlog_10_ lung CFU) between unvaccinated and vaccinated mice was statistically significant. The CC strains were ranked from most protected (CC037, Δlog_10_ CFU = 1.6) to least protected (CC040, Δlog_10_ CFU = –0.6) (Figure 1B, bottom). BCG vaccination exacerbated subsequent Mtb infection in the lung for 2 CC strains (CC003 and CC040), as had been observed in the NZO founder strain (19). Importantly, BCG led to significant reductions in lung CFU in only 13 of the 24 CC strains.

We next asked whether the protective effect of BCG vaccination correlated with the inherent ability (i.e., the unvaccinated state) of each CC strain to control Mtb replication. We categorized the 24 CC strains relative to C57BL/6J mice. The lung CFU of unvaccinated mice defined the inherent susceptibility or resistance to Mtb. Whether each CC strain was protected was based on the Δlog_10_ lung CFU (relative BCG-induced protection). The plot was centered on C57BL/6J mice, which represented a median strain in terms of lung susceptibility and protection and divided the 24 CC strains into 4 different groups (Figure 1C). Based on lung susceptibility and protection, there was no significant correlation between intrinsic susceptibility of the mouse strain and protection conferred by BCG at this time point (Pearson *r* = 0.104, *P* = 0.62).

The same analysis was applied to splenic Mtb CFU as a measure of systemic infection. As for the lung, the CC population was protected by BCG (Figure 1D). Protection in the spleen extended from most protected (CC026, Δlog_10_ CFU = 2.56) to least protected (CC045, Δlog_10_ CFU = 0.13) (Figure 1E). BCG significantly protected against systemic infection in 17 of the 24 CC strains (Figure 1E). There was also not enough evidence to suggest correlation between susceptibility and protection in the spleen (Pearson *r* = 0.089, *P* = 0.67). Interestingly, while C57BL/6 mice were a median strain in the lung analysis, C57BL/6 mice had among the highest spleen CFU in primary infection but were among the best protected strains following BCG vaccination (Figure 1F). The skewing illustrates that vaccination studies using C57BL/6 mice was not representative of genetically diverse populations (Figure 1F). Among the CC strains, protection in the lung and the spleen were moderately correlated (Pearson *r* = 0.513, *P* = 0.0087).

In follow-up studies, 13 of the 24 CC strains were retested to confirm the initial phenotype, (Supplemental Table 1; supplemental material available online with this article; https://doi.org/10.1172/JCI167762DS1). With the exception of CC023, we were able to confirm our findings from the first round of analysis. Thus, using 24 CC strains, with diverse genotypes, we found that less than 50% of the strains were protected by BCG. Protection did not correlate with the intrinsic susceptibility of the strain to Mtb infection. Finally, the lack of correlation between the reduction in lung versus spleen highlights the importance of tissue-specific immunity and the problems inherent in protecting the lung against infection. We next assessed how BCG vaccination altered the histological appearance of TB lung lesions.

### BCG modifies the lung lesions caused by Mtb in a strain-specific manner.

**Figure 2:**
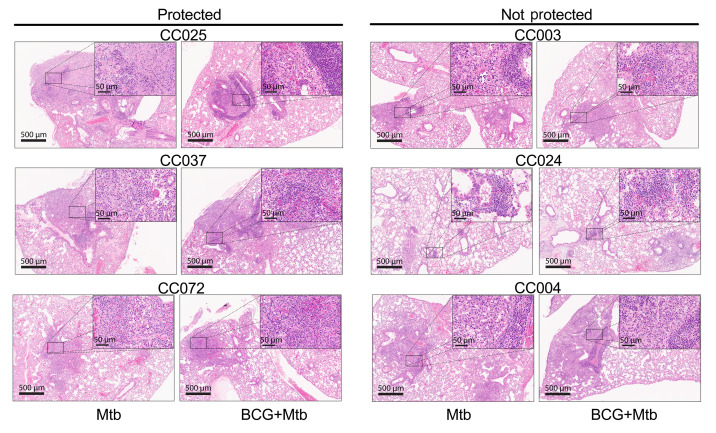
Lung histology of CC mice after BCG vaccination and Mtb challenge. Representative lung histology of 3 BCG-protected CC strains (CC025, CC037, CC072) (left), and 3 unprotected CC strain (CC003, CC004, CC024) (right). Original magnification, ×40 (inset, ×400). Scale bars: 500 μm (inset, 50 μm).

We assessed how BCG vaccination modified lung lesion development after Mtb infection in 19 different CC strains plus C57BL/6 mice. A board-certified veterinarian pathologist, blinded to vaccination status of each strain, examined the lung for necrosis, neutrophilic and lymphocytic infiltrates, and lesion size and number. In 15 of the 20 strains, the vaccinated group was correctly identified based on reduced severity of the histopathology. In some protected CC strains, increased lymphocytic infiltration and reduced necrosis were observed after vaccination (Supplemental Table 2). CC025 (Figure 2, top left) and CC037 (Figure 2, middle left) were both susceptible to Mtb infection, and the infected lung showed innate cells and tissue necrosis. BCG vaccination protected these mice by promoting lymphocyte recruitment, and, in CC025, BCG vaccination reduced necrosis. However, lung CFU reductions associated with BCG vaccination were not always accompanied with an improved histological appearance. Despite a significant reduction in lung CFU after BCG, no discernable histopathological differences were observed between unvaccinated and vaccinated CC072 mice, and both groups had nonnecrotizing lymphocyte–rich granulomas (Figure 2, bottom left).

Two of the 11 nonprotected CC strains, CC003 (Figure 2, top right) and CC024 (Figure 2, middle right), had no discernable histological differences in granuloma structure, content, or severity between control and BCG vaccination when evaluated blindly. In these 2 strains, lesions were generally small to medium in size, nonnecrotizing, with abundant perivascular and peribronchiolar lymphocytes. Given the intermediate susceptibility of the CC003 strain and BCG’s inability to alter lung CFU or histology, we suggest that BCG did not change strain CC003’s innate or adaptive immunity to Mtb. Likewise, resistant CC024 mice were unprotected by BCG vaccination, and its lung lesions were unaltered, suggesting that BCG vaccination failed to enhance the natural resistance of CC024 to Mtb. In contrast, CC004 (Figure 2, bottom right) had discernable histopathological changes when controls and BCG vaccinees were evaluated. The differences attributable to BCG vaccination included the greater lymphocytic infiltration with denser perivascular and peribronchiolar lymphocytes. The altered histology shows that BCG modulated the immunity to Mtb, despite being unable to control bacterial replication. Together, these data show that the host genetic background affects the granuloma content, structure, and quality of the immune response to BCG and Mtb challenge in a manner that is not wholly predictable.

### BCG-protected CC strains have an altered lung microenvironment after Mtb infection.

The Mtb-infected lung is a complex environment of resident and recruited cells that produce inflammatory mediators and counter-regulatory antiinflammatory signals. We hypothesized that BCG vaccination would prime an immune response that would generate a lung microenvironment that would be less conducive to bacillary replication upon Mtb challenge. To identify immunological features that correlated with protection across diverse genotypes, cytokine levels in lung homogenates from unvaccinated and BCG-vaccinated C57BL/6 or CC mice were measured 4 weeks after infection.

**Figure 3:**
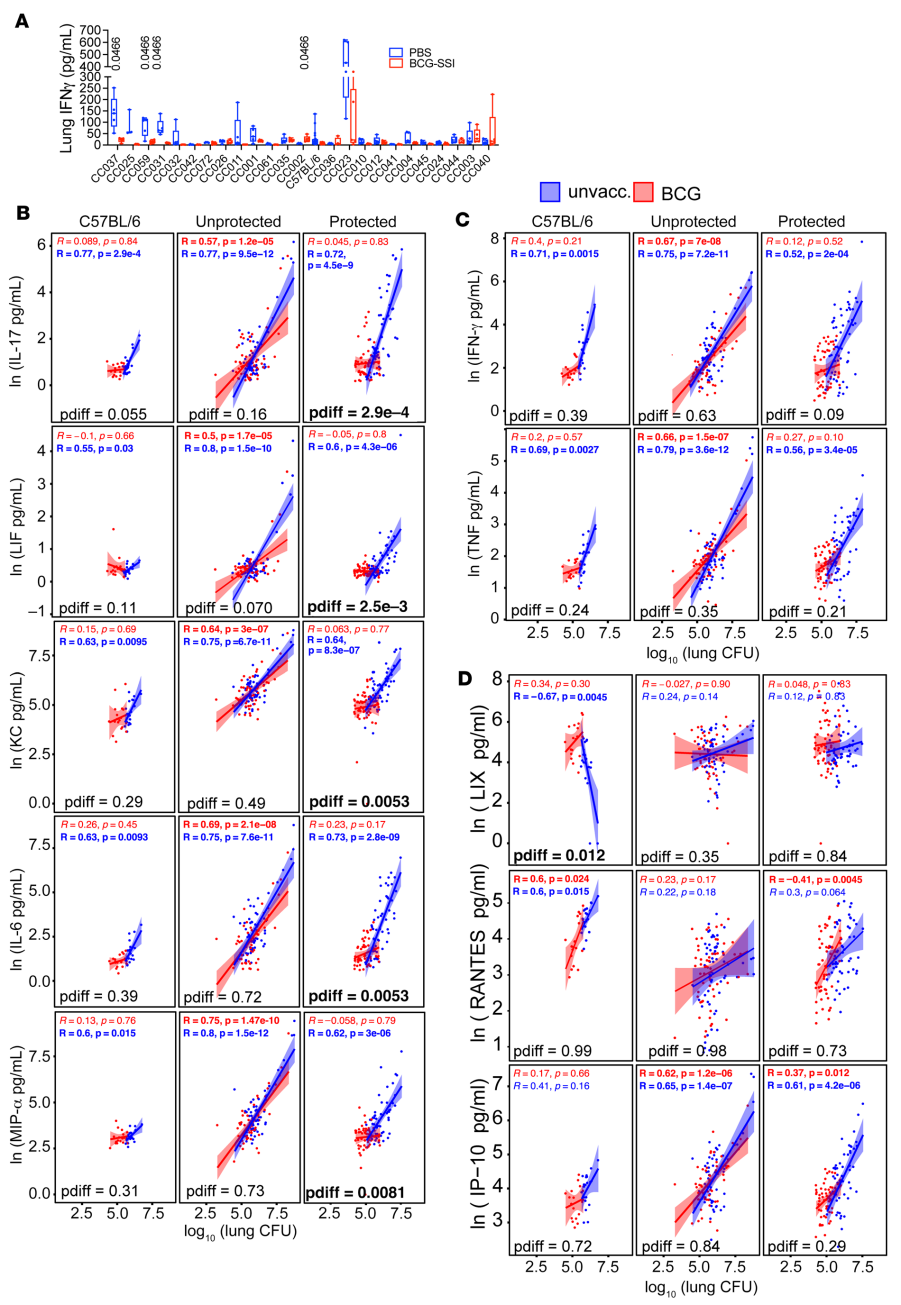
BCG-protected CC mice have an altered lung cytokine environment after Mtb infection. (**A**) Lung homogenate IFN-γ levels from BCG vaccinated or unvaccinated CC mice 4 weeks after infection, represented as box-and-whisker plots, with bounds from 25th to 75th percentile, median line and whiskers ranging from minimum to maximum. A 2-way ANOVA was performed using the original FDR method of Benjamini and Hochberg. FDR values indicated above bars. (**B**–**D**) Pearson correlation between lung CFU and ln transformed (**B**) IL-17, LIF, KC, IL-6, and MIP-1α; (**C**) IFN-γ and TNF; and (**D**) LIX, RANTES, and IP-10 comparing unvaccinated and BCG vaccinated C57BL/6 mice, and unprotected or protected CC mice. R values indicate the Pearson correlation coefficients for unvaccinated (blue) and BCG (red) groups, with the accompanying *P* values indicate the Benjamini-Hochberg corrected significances of those correlations. Within each category of C57BL/6, not-protected CC mice, and protected CC mice, the correlations between BCG vaccinated and unvaccinated mice were compared using a Fisher’s z transformation of the correlation coefficients followed by a z test, and the significance of the difference in the correlations is reported as a Benjamini-Hochberg corrected pdiff. Significant *P* values are in bold.

Given the critical role of IFN-γ in Mtb control, its levels in control and BCG-vaccinated mice were compared. In C57BL/6 mice and many of the protected CC strains, including CC037, CC025, CC059, CC031, and CC011, IFN-γ concentrations in BCG-vaccinated mice were reduced compared with unvaccinated mice (Figure 3A). However, this pattern did not hold for all protected strains. No changes in IFN-γ levels were detected in the CC032, CC042, CC072, and CC026 strains despite being protected by BCG. Thus, when considered alone, neither the absolute level of IFN-γ nor its change after vaccination can explain protection.

We next asked if there was a correlation between the different cytokines in the lung homogenate and CFU in either unvaccinated or BCG-vaccinated mice, and if so, whether it differed among protected CC strains, unprotected CC strains, and C57BL/6 mice. A similar pattern existed for several cytokines, including IL-17, LIF, KC, IL-6, MIP-1α, IFN-γ, and TNF (Figure 3, B and C). A significant correlation existed between the lung cytokine and CFU for unvaccinated and vaccinated mice among the unprotected CC strains. Among the protected CC strains, a significant correlation was found only for the unvaccinated mice. The protected C57BL/6 strain followed the same pattern as protected CC strains. Comparing the Pearson correlation coefficients for unvaccinated and vaccinated mice within each group indicated that, for several cytokines, the correlation coefficients significantly differed between unvaccinated and vaccinated mice in protected CC strains but not unprotected CC strains. Interestingly, the differences between the unvaccinated and vaccinated correlation coefficients were not significant for IFN-γ and TNF (pdiff = 0.09 and 0.21, respectively). This indicates that BCG vaccination changed the relationship between bulk lung cytokine and bacterial burden in protected CC strains for IL-17, LIF, KC, IL-6, and MIP-1α (Figure 3B) more so than for IFN-γ and TNF (Figure 3C). Meanwhile, an unchanged cytokine-CFU relationship for these cytokines was a signature of the unprotected phenotype.

A few cytokines had different trends (Figure 3D). LIX (CXCL2) was negatively associated with lung CFU in unvaccinated — but not in BCG-vaccinated — C57BL/6 mice, and it was the only cytokine in the panel for which vaccination significantly changed the correlation coefficient in C57BL/6 mice (pdiff = 0.012). In contrast, neither unprotected nor protected CC mice had a correlation between LIX and CFU. RANTES was also unique in that it strongly correlated with lung CFU in unvaccinated and vaccinated C57BL/6 mice and in vaccinated protected CC strains, but not in unprotected CC strains. Finally, IP-10 correlated with CFU in unvaccinated and vaccinated CC mice of both protection groups, but not with C57BL/6 mice. The LIX and IP-10 results further support the idea of C57BL/6 mice being an outlier.

Across the board, what is largely consistent is that the relationship between cytokines and bacterial burden did not change in unprotected CC strains, while this relationship did change in protected CC mice. This relationship was mostly unchanged statistically in C57BL/6 mice, though this could be due to insufficient power to detect differences in a single strain. These data show how differences in the host genetic background result in varied relationships between lung homogenate cytokines and lung bacterial burden.

### Genetic background, not BCG vaccination, largely determines T cell–subset distribution in the lung after Mtb infection.

**Figure 4:**
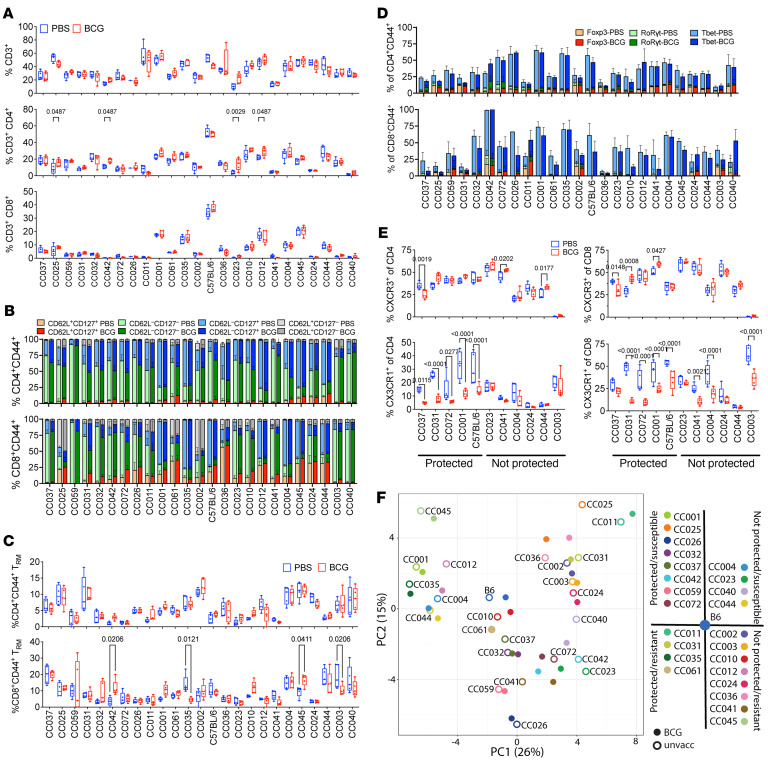
T cell populations in protected and unprotected CC mice following Mtb infection. (**A**) Frequency of CD3^+^, CD3^+^CD4^+^ and CD3^+^CD8^+^ T cells in the lungs of unvaccinated and BCG-vaccinated CC mice 4 weeks after infection. (**B**) Proportion of memory CD4 and CD8 T cells in the lungs of either unvaccinated or BCG-vaccinated CC mice 4 weeks after infection, as defined by CD44^+^ and various combinations of CD62L and CD127 expression; CD62L^+^CD127^+^ (central memory), CD62L^–^CD127^–^ (effector), CD62L^–^CD127^+^ (effector memory). (**C**) Frequency of resident memory CD4 and CD8 T cells in the lungs of either unvaccinated or BCG-vaccinated CC mice 4 weeks after infection, as defined by CD44^+^CD103^+^CD69^+^. (**D**) Proportion of Th1, Treg and Th17 cells in the lungs of either unvaccinated or BCG-vaccinated CC mice 4 weeks after infection, as defined by CD44^+^ and Tbet^+^ (Th1), Foxp3^+^ (Treg) or RoRγt (Th17) expression. (**E**) Frequency of CD4 and CD8 T cells expressing either CXCR3 or CX_3_CR1 in the lungs of either unvaccinated or BCG-vaccinated CC mice 4 weeks after infection. (**F**) PCA of the T cell–phenotyping data. Each point represents the average of 5 mice within a given mouse strain that are either BCG vaccinated or unvaccinated. Open symbols, unvaccinated; closed symbols, BCG vaccinated. Each color represents a different mouse strain. The unvaccinated symbol is labeled with the CC strain name. The legend is modeled after the quadrants in Figure 1C. (**A**–**E**) The data represent the mean ± SEM from 1 experiment (*n* = 5/group). 2-way ANOVA with the Benjamini and Hochberg multiple comparison method. The FDR was set to 0.05 and the numbers in the figures are the *q* value. Quantitative data in panels **A**, **C**, and **E** are represented as box-and-whisker plots, with bounds from 25th to 75th percentile, median line and whiskers ranging from minimum to maximum.

We hypothesized that changes in the frequency of T cell subsets would correlate with protection induced by BCG vaccination. A multi-parameter flow cytometry panel was used to identify and enumerate different immune subsets (Supplemental Figure 1). We quantified B cells, CD4 and CD8 T cells, and mucosal-associated invariant T (MAIT) cells (Figure 4A and Supplemental Figure 2). Memory and effector T cell populations were defined as central memory (T_CM_, CD44^+^CD62L^+^CD127^+^), effector memory (T_EM_, CD44^+^CD62L^–^CD127^+^), effector (T_Eff_, CD44^+^CD62L^–^CD127^–^), and resident memory (T_RM_, CD44^+^CD103^+^CD69^+^) (Figure 4, B and C). CD4 T cell subsets were defined in all CC strains based on their expression of the transcription factors Foxp3, Tbet, Gata3, and RORγt to identify Treg, Th1, Th2, and Th17 cells, respectively (Figure 4D). Gata3 expression had universal low expression and was not analyzed further. For the other T cell subsets, we observed tremendous strain-to-strain variation. In general, BCG vaccination did not modulate the distribution of T cell subsets nor was there any correlation between the relative frequency of these T cell subsets and the outcome of BCG vaccination. Thus, the frequency of different T cells subsets in the lungs of CC mice after Mtb infection was largely determined by each strain’s genetic background and not their immunization status.

We analyzed CXCR3 and CX_3_CR1 expression in a limited number of CC strains, as it has been used to discriminate between lung parenchymal and circulating T cells (22). Like other markers, CXCR3 expression varied between different CC strains but did not correlate with vaccination status or susceptibility. CXCR3 expression was not detected in CC003 mice (Figure 4E). CC003 has the PWK allele of the CXCR3 gene, and we were unable to detect CXCR3 expression by PWK mice (data not shown). The inability of the anti-CXCR3 mAb to detect the PWK allele of CXCR3 could be the consequence of a haplotype-specific mutation (23). Interestingly, a reduction in CX3CR1^+^ CD4 and CD8 T cells was observed in several protected CC strains but not unprotected strains (Figure 4E). This pattern mirrors the reduction in IFN-γ levels observed in some protected CC mice (Figure 3), suggesting that these changes reflect Th1 modulation.

Finally, we used principal component analysis to holistically visualize the variation in the T cell phenotype (Figure 4E). Strikingly, BCG vaccination had only a small effect in altering T cell phenotype compared with the large effect of CC strain differences. Together, these data indicate that genetic differences between CC strains had a larger role in determining the T cell response recruited to the lung after TB than BCG vaccination did. Therefore, we next determined whether functional differences existed among the T cell subsets in protected versus nonprotected CC strains.

### Cytokine production by T cells differ in CC strains after BCG vaccination and Mtb infection.

**Figure 5:**
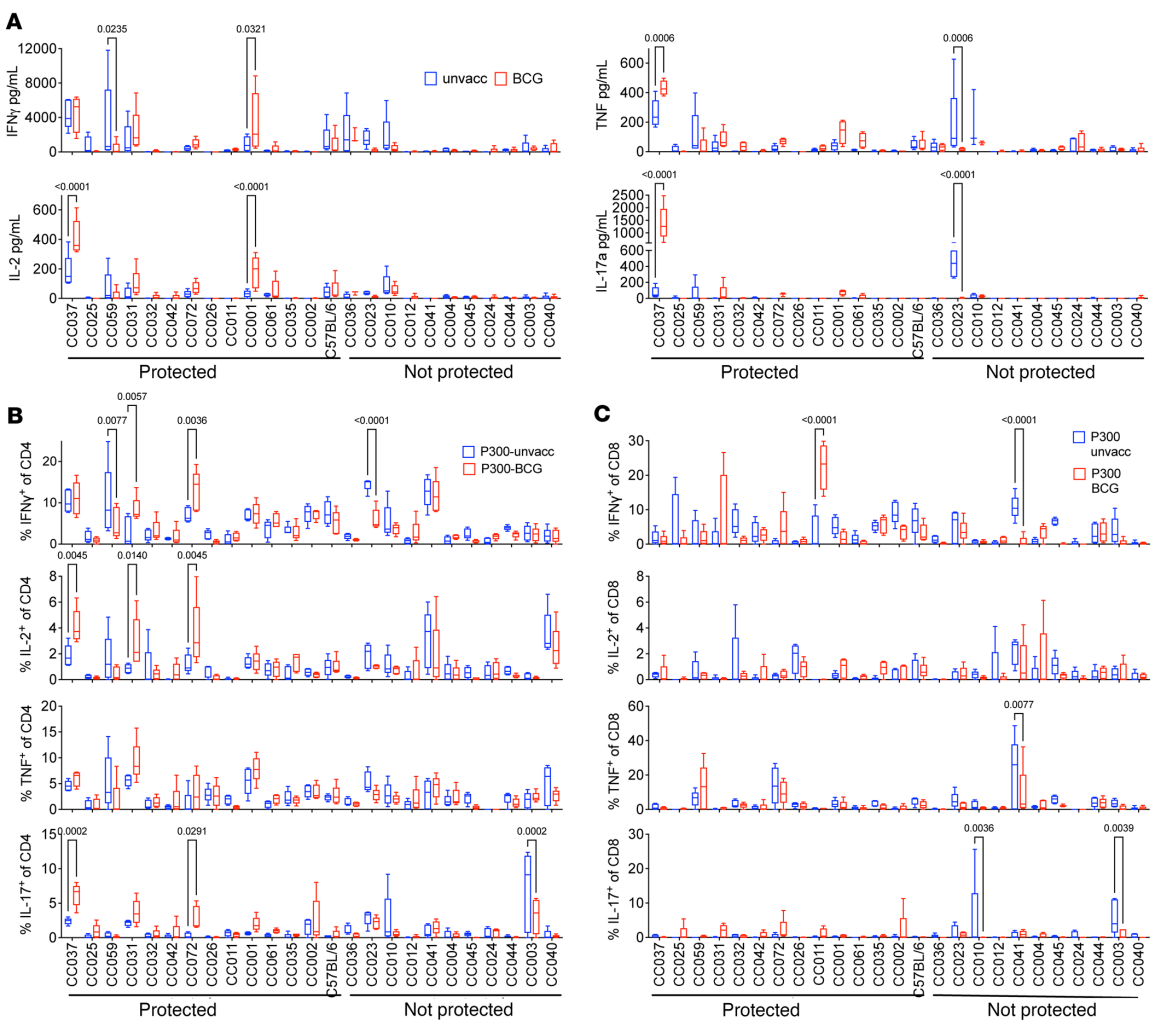
Cytokine responses in CC strains following Mtb infection. (**A**) Lung cells 4 weeks after infection were stimulated for 24 hours with the MTB300 peptide pool. IFN-γ, IL-2, TNF and IL-17A were measured in the supernatants. (**B** and **C**) Lung cells were stimulated for 5 hours with the MTB300 peptide pool. Frequencies of CD4 or CD8 T cells that produced IFN-γ, IL-2, TN,F or IL-17 was determined using intracellular cytokine staining. (**A**–**C**) The data represent the mean ± SEM from 1 experiment (*n* = 5/group). 2-way ANOVA with the Benjamini and Hochberg multiple comparison method was used to determine significance. Quantitative data are represented as box-and-whisker plots, with bounds from 25th to 75th percentile, median line and whiskers ranging from minimum to maximum.

As cytokines produced by T cells, including IFN-γ and TNF, are important in Mtb control, we hypothesized that changes in T cell function would be associated with BCG-mediated protection. To measure function, we stimulated lung mononuclear cells (MNC) from each mouse with anti-CD3 or the MTB300 megapool, which contains 300 peptides representing epitopes from 90 Mtb proteins that are frequently recognized by human CD4 T cells and murine T cells (24, 25). Twenty-six cytokines and chemokines in culture supernatants were measured 24 hours after stimulation. Lung MNC from unvaccinated or BCG-vaccinated Mtb-infected C57BL/6 mice did not differ in their secretion of cytokines or chemokines following MTB300 stimulation. In contrast, IFN-γ, TNF, IL-2 or IL-17, were differentially produced by some unvaccinated versus BCG-vaccinated CC strains (Figure 5A). Interestingly, the lung MNC from BCG vaccinated CC037, CC031, CC72, and CC001 strains produced more IL-17 than their unvaccinated controls. In contrast, none of the unprotected strains produced significant amounts of IL-17, except for CC023, which produced less IL-17 after BCG vaccination.

In parallel, we measured intracellular levels of IFN-γ, IL-2, IL-17, TNF, IL10, IL-4, and CD107 surface expression by CD4 and CD8 T cells (Supplemental Figure 3). BCG modified CD4 T cell cytokine responses in C57BL/6 mice and several CC strains (Figure 5B). Among CD8 T cells, BCG vaccination primarily altered the IFN-γ response to MTB300 (Figure 5C). Patterns of cytokine production were observed in BCG vaccinated CC mice that were not observed in C57BL/6 mice. For example, while BCG vaccination did not alter the frequency of IFN-γ^+^ or TNF^+^ CD4 T cells in CC037 mice, it did increase IL-2 and IL-17 responses. BCG vaccination of CC031 mice increased the frequency of CD4 T cells producing IFN-γ, IL-2,and TNF, but not IL-17. Finally, CD4 T cells from the BCG vaccinated CC072 mice had increased IFN-γ and IL-2 but not TNF or IL-17. No discernable differences in cytokine responses were observed between vaccinated and unvaccinated mice in most of the nonprotected CC mice, except for a decrease in IFN-γ responses in CC023. Thus, the cytokine responses in both control and BCG-vaccinated CC mice differ greatly from the classic C57BL/6 model. Increased cytokine responses after T cell stimulation were more likely to be detected in protected CC strains. Moreover, BCG vaccination led to quantitative and qualitative changes in the type of T cell responses that persisted after Mtb challenge.

### Identifying correlates of protection using multivariate approaches.

**Figure 6:**
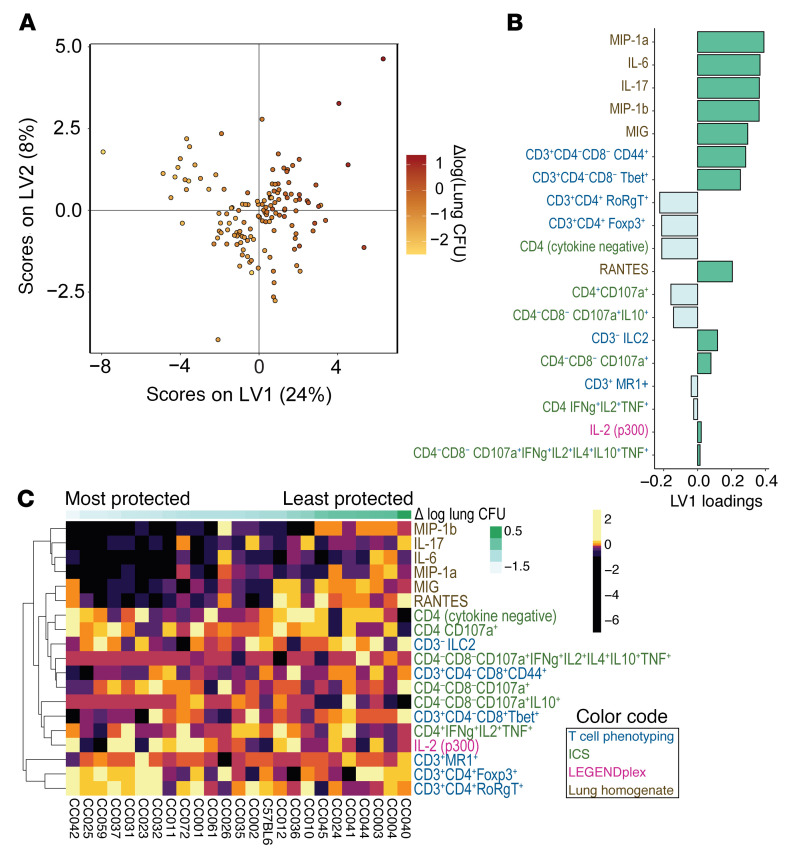
Multivariate correlates of protection across all 24 CC strains. (**A**) A PLSR model was performed to associate BCG-induced changes in lung immune features with BCG-induced changes in lung CFU. Each data point represents a vaccinated mouse that has the mean of the unvaccinated mice from the same strain subtracted from it. The scores of the top 2 latent variables of the model are shown. The color of each data point represents the change in lung CFU induced by BCG vaccination. The percentages of the variance in the lung features captured by each latent variable are shown as percentages in the axes. (**B**) The bar graph depicts the loadings of the first latent variable of the PLSR model (**A**). Positive loadings (i.e., on the positive x-axis in the scores plot) indicate immune features that increase in BCG-vaccinated mice, which generally indicates an association with worse protection. Negative loadings indicate immune features that increase in mice protected by BCG. (**C**) The heatmap illustrates all features selected included in the model, indicating their importance in associating CC strains with the lung protection continuum. Colors are representations of z-scored features, with a different color representing each 10% quantile of the data. Columns are averages of the CC strains (*n* = 5 for most cases). Feature names are colored based on the data set from which they come. (**B** and **C**), features from the ICS data set indicate production of the cytokines listed and no production of any measured cytokines not listed.

We turned to multivariate approaches to discern how BCG-induced immune changes in the lung holistically lead to reductions in lung CFU in CC strains. A multivariate partial least squares regression (PLSR) model successfully predicted lung CFU changes from BCG-induced immune changes as validated in a 5-fold cross validation framework (Figure 6A and Supplemental Figure 4A). The features selected through elastic net as being most correlated with lung CFU belonged to all 4 data sets (Figure 6, B and C). By plotting the selected features for all CC strains, ordered from most to least protected, it is apparent that the protected strains all had BCG-induced reductions in several inflammatory mediators such as MIG, MIP-1α and -1β, IL-6, RANTES, and IL-17. These decreases could reflect reduced infection, while increased inflammatory cytokines in the unprotected strains could signify unimpeded infection. Protected strains also had greater vaccination-induced increases in activated CD4 T cells expressing RORγt or FoxP3, which could be Th17 and Treg cells, respectively. Contrarily, protected strains had decreased activated CD4^–^CD8^–^ T cells expressing T-bet. Importantly, stimulated IL-2 production, unlike several other cytokines, tended to increase in protected strains. Thus, BCG vaccination led to changes in T cell immunity and in the immune environment of the lung after Mtb infection in CC genotypes that were protected by BCG vaccination.

### Immune responses in CC mice differ across protection and susceptibility categories.

**Figure 7:**
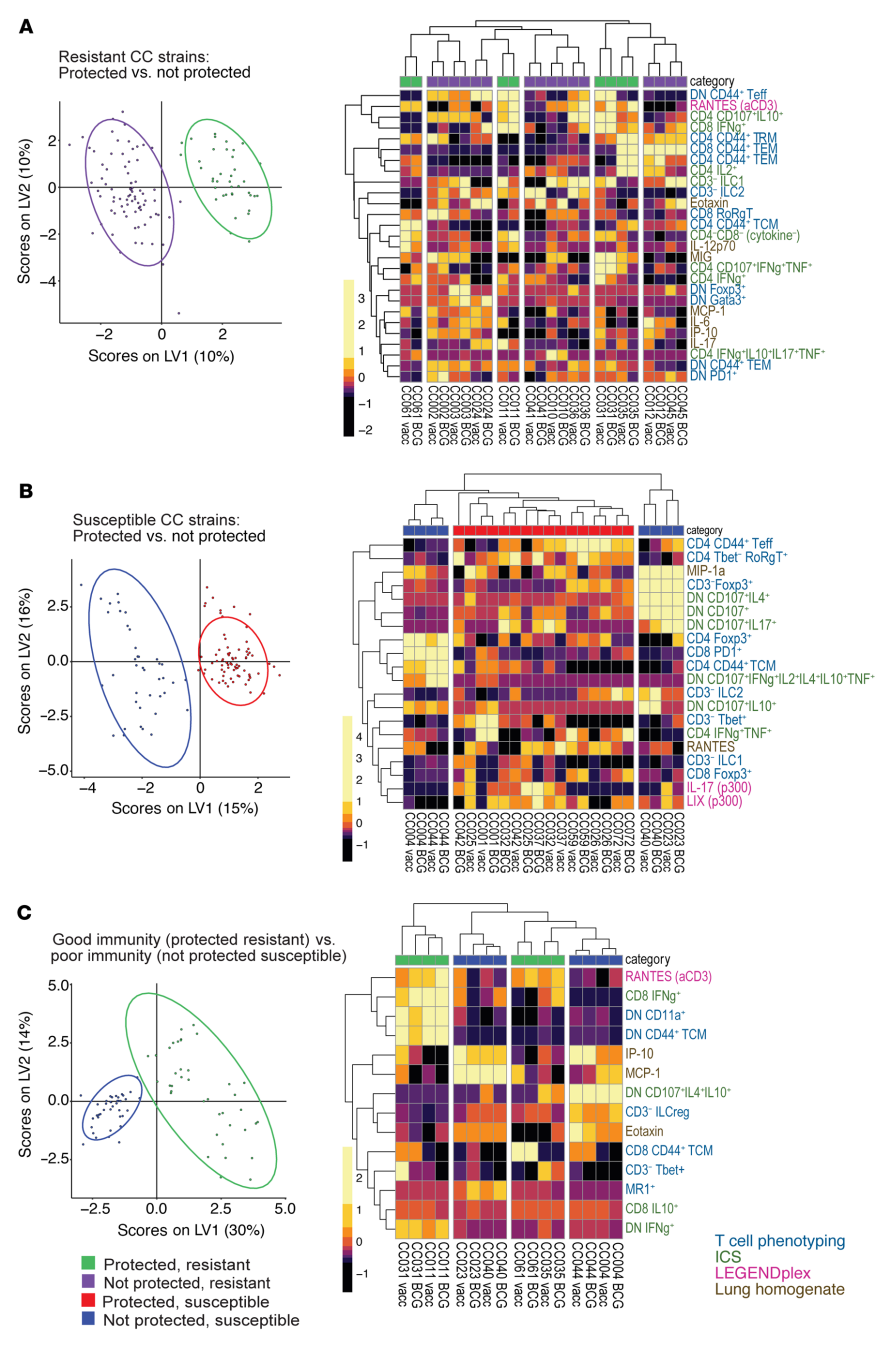
Identifying signatures of protection in categories of CC strains. Scores plot (left) and heatmaps (right) of selected features for PLSDA models comparing (**A**) resistant CC strains (**B**) susceptible CC strains and (**C**) protected resistant and non-protected susceptible mice (i.e., good and poor immunity, respectively). Each dot represents a single mouse. The 2 colors in the scores plots signify the 2 classes being compared. An ellipse is drawn around the 95% CI for each class. Percent of variance of the immune feature data are shown on the axes. Heatmaps show features that were most important for distinguishing the 2 classes. Colors are representations of z-scored features. Columns are averages of the mice in the CC strains, with vaccinated and unvaccinated mice being separated (*n* = 5 for most cases). Feature names are colored based on the data set from which they come (blue, T cell phenotyping; brown, lung homogenate cytokines; green, ICS; magenta, LEGENDplex). The legend references the protection/susceptibility quadrant (Figure 1C).

**Figure 8:**
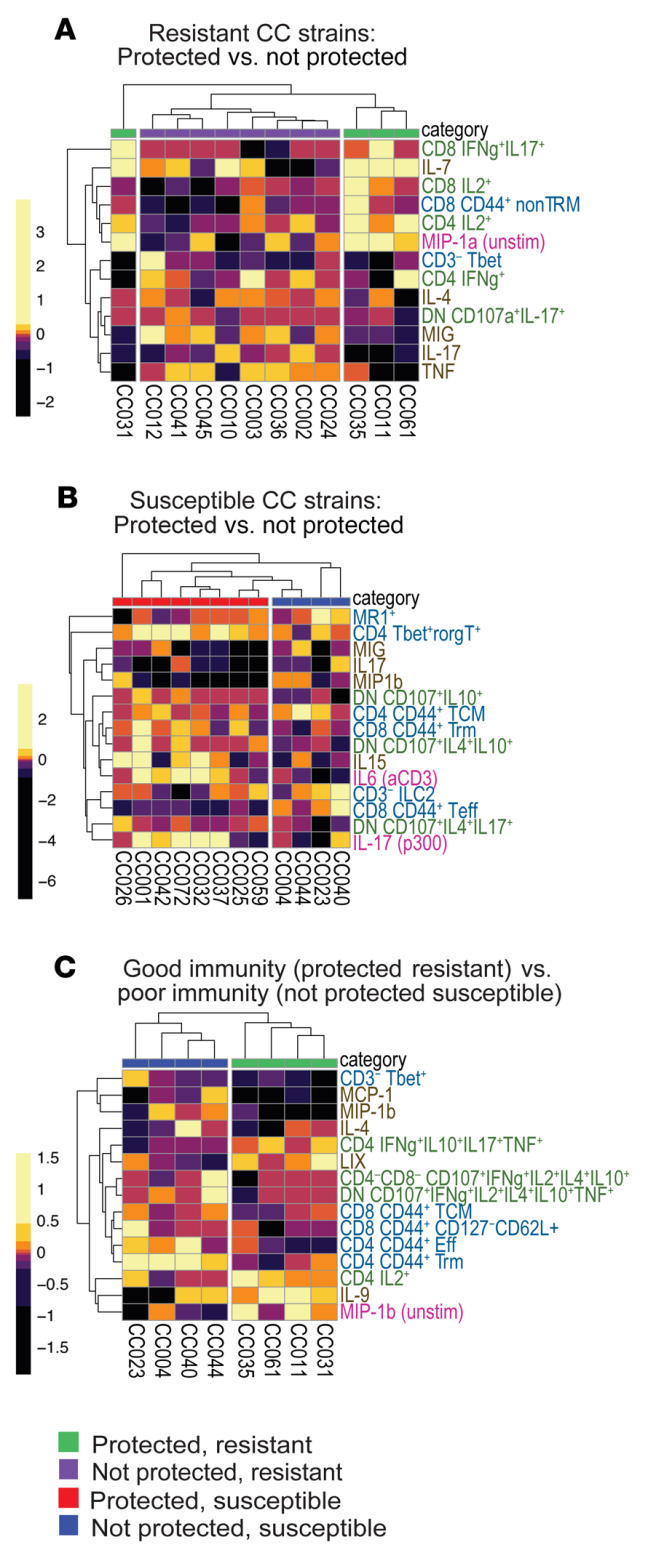
Identifying vaccine-induced signatures of protection after BCG vaccination in CC strains. PLSDA models performed as in Figure 7, except that features are the changes induced by BCG vaccination as in Figure 6. (**A**–**C**) Heatmaps of selected vaccine-induced features for PLSDA models comparing (**A**) resistant CC strains (**B**) susceptible CC strains and (**C**) protected resistant and non-protected susceptible mice (i.e., good and poor immunity, respectively). Heatmaps show features that were most important for distinguishing the 2 classes. Colors are representations of z-scored features. Columns are averages of the mice in the CC strains, with vaccinated and unvaccinated mice being separated (*n* = 5 for most cases). Feature names are colored based on the data set from which they come blue, T cell phenotyping; brown, lung homogenate cytokines; green, ICS; magenta, LEGENDplex). The legend references the protection/susceptibility quadrant (Figure 1C).

While the PLSR model highlights potential correlates of protection across all genotypes, it may miss correlates that only apply to certain subsets of CC strains. We hypothesized that vaccination may protect naturally resistant strains and that susceptible strains may be protected by different mechanisms. To test this, we created separate partial least squares discriminant analysis (PLSDA) models for resistant and susceptible CC strains and examined the features that best separated the BCG-protected and nonprotected strains in each of these categories. (Figure 1C). The data used for the models were processed the same way as in the PLSR models, although we also examined the vaccinated and unvaccinated mice separately (Figure 7, A–C) in addition to vaccine-induced differences (Figure 8, A–C). All models could predict the 2 compared categories accurately, suggesting that there were multivariate signatures for these categories (Supplemental Figure 4B). Heatmaps of each model’s selected features were hierarchically clustered both by feature and CC strain and used to characterize the facets of the immune response distinguishing the groups. Our first analysis compared resistant CC strains that were protected by BCG (*n* = 4) or not (*n* = 8), based on lung CFU. As described earlier (Figure 4), the unvaccinated and vaccinated mice of each CC strain were very similar and clustered together when the model-selected features were hierarchically clustered, verifying that the genetic background was an important determinant of outcome (Figure 7A). The fact that both protected and unprotected mice were split into multiple clusters and that there were no clear individual features distinguishing the categories shows that these strains have unique immune responses. In contrast, the vaccine-induced model comparing protected and nonprotected resistant strains yielded more univariate trends in addition to the strong multivariate signature (Figure 7B). Most prominently, CD4 T cells making IL-2 were increased in protected strains, as were lung IL-7 and MIP-1α, while other lung cytokines like TNF and IL-17 decreased (Figure 7B). This shows that, while the broad immune response in resistant mice is diverse, narrowing in on vaccine-induced changes reveals a more uniform route to protection.

We next analyzed CC strains that were inherently more susceptible than C57BL/6 mice and were either protected by BCG (*n* = 4) or not (*n* = 8) (Figure 7C). This yielded 2 clear subclusters defining susceptible, nonprotected CC strains. The CC004 and CC044 subcluster expressed high levels of T_CM_, CD4^+^Foxp3^+^, CD8^+^PD-1^+^, and polyfunctional CD4^–^CD8^–^ T cells. The other cluster had high levels of lung MIP-1α and CD3^–^Foxp3^+^ T cells and fewer polyfunctional CD4^–^CD8^–^ T cells. The vaccination model (Figure 8A) shared several features with the model including all mice (Figure 7C). However, the best distinguishing features were unique to the vaccination model and included increased amounts of stimulated IL-6, IL-17A, and IL-15 in protected mice compared with unprotected mice. Common to both the resistant and susceptible mouse models was the fact that overall lung IL-17 decreased due to BCG vaccination in protected mice, while T cell populations making IL-17 increased. It is also notable that many of the best distinguishing features are cytokine-based rather than T cell phenotype–based. Overall, our analysis identified features that distinguished protected from nonprotected mice in both resistant and susceptible backgrounds.

Finally, to identify features that differed between restrictive and permissive immune responses to Mtb, we compared susceptible CC strains that were unprotected by BCG with resistant CC strains that were protected by BCG. Unsurprisingly, there was a clear separation of categories in the model with all mice. Poor immunity CC strains tended to have high levels of inflammatory monokines (Figure 8B). Good immunity CC strains had high levels of RANTES after anti-CD3 stimulation. A subset of good immunity strains, including CC031 and CC011, had high frequencies of IFN-γ–producing CD8 T cells and various subsets of CD4^–^CD8^–^ T cells that produced IFN-γ or expressed CD11a. In contrast, a subset of poor immunity strains had high levels of polyfunctional CD4^–^CD8^–^ T cells and ILCregs. The protection model revealed similar features with the addition of increased activated CD4 T_RM_ cells after BCG vaccination in poor immunity but not good immunity CC strains (Figure 8C). We found immune features that could be identified using PLSDA, which could categorize the inherent susceptibility and response to BCG of CC strains following Mtb infection. Looking at more refined subgroups of CC strains allowed us to find features that defined subclasses of protected and nonprotected strains and highlight features that did not emerge in the PLSR model, such as non-T_RM_ effector and T_CM_ populations.

### Th1/17 cells detected in a subset of CC mice that are protected by BCG vaccination.

**Figure 9:**
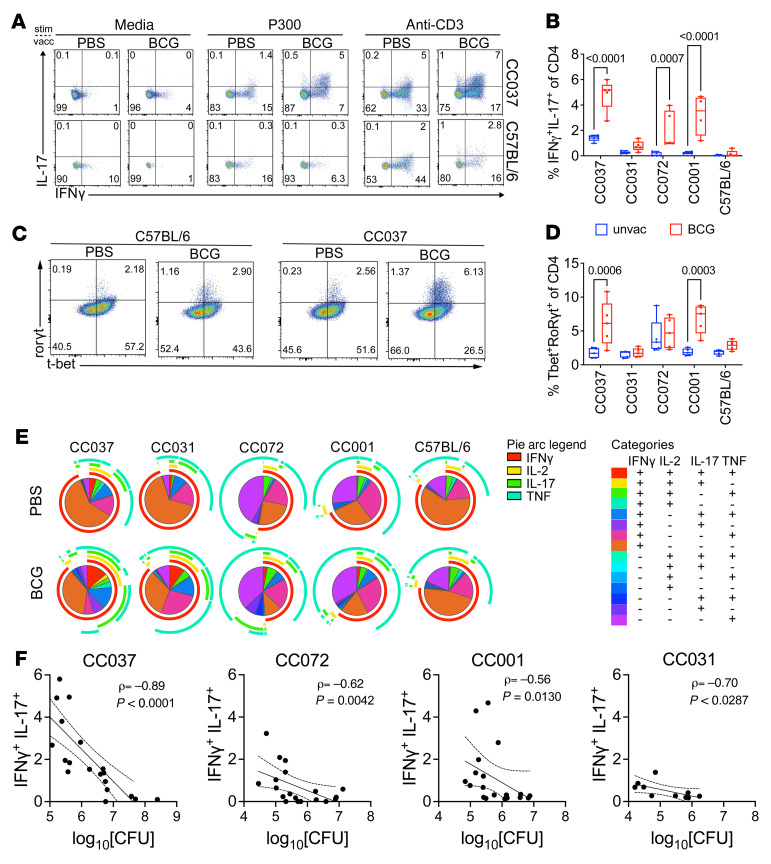
Th1/17 cells correlate with BCG-mediated protection in a subset of protected CC mice. (**A**) 4 weeks after infection, lung cells were stimulated with anti-CD3 mAb, MTB300 megapool,or nothing (media). Representative flow plots of IFN-γ and IL-17–producing CD4 T cells from CC037 and C57BL/6 mice. (**B**) Frequencies of CD4 T cells that expressed both IFN-γ and IL-17. (**C**) Representative flow plots of T-bet and RORγt expressing CD4 T cells from CC037 or C57BL/6 mice. (**D**) Frequencies of CD4 T cells that expressed both T-bet and RORγt. (**E**) Proportion of CD4 T cells that express combinations of IFN-γ, TNF, IL-2, or IL-17 in unvaccinated or BCG vaccinated mice 4 weeks after Mtb infection, generated using SPICE (41). (**F**) Correlation of IFN-γ^+^IL-17^+^ cells and lung CFU from unvaccinated or BCG vaccinated CC037, CC072, CC001, and CC031 mice 4 weeks after Mtb infection. Unvaccinated and BCG vaccinated mice are combined for the correlation analysis. Spearman ρ and P value are shown. Data are pooled from 2 experiments except for CC031. Data are representative of 2 independent experiments (n = 5 mice/group). 1-way ANOVA corrected with the Benjamini and Hochberg multiple comparison method (FDR = 0.05).

T cells expressing IL-17 and RORγt emerged as important correlates in several of the multivariate protection models. Vaccination significantly enhanced IL-17 responses univariately in the CC037, CC001, CC072, and CC031 strains, which were all protected by BCG (Figure 5, A and B). As the CC strains that produced IL-17 after antigen stimulation also had strong IFN-γ responses, we considered whether these T cells produced both IFN-γ and IL-17. After stimulation with MTB300, some CD4 T cells in BCG-vaccinated CC037 mice produced both IFN-γ and IL-17 (Figure 9A). Such IFN-γ/IL-17–producing CD4 T cells were not detected in C57BL/6 mice, regardless of vaccination status. BCG vaccination significantly increased the frequency of IFN-γ/IL-17–producing CD4 T cells in CC037, CC072, and CC001 mice (Figure 9, A and B). Further analysis identified BCG primed CD4 T cells from CC037 and CC001 to coexpress both Tbet and RORγt after Mtb infection (Figure 9, C and D), suggesting that these were Th1/17 cells.

We next measured T cell polyfunctionality. In addition to IFN-γ and IL-17, an increase in the proportion of CD4 T cells producing IL-2 or TNF was detected (Figure 9E), indicating that CD4 T cell polyfunctionality was increased in BCG-vaccinated mice. A detailed analysis of the different polyfunctional populations revealed different distributions of Th1/17 cells within each CC strain. IFN-γ^+^IL-2^+^TNF^+^IL-17^+^ cells were significantly increased in CC037 following BCG vaccination, while frequency of IFN-γ single–positive and IFN-γ^+^TNF^+^ CD4 T cells were diminished (Supplemental Figure 5A, red versus purple boxes). Similarly, IFN-γ^+^TNF^+^IL-17^+^ cells were increased following BCG vaccination in CC072 and CC001 strains (Supplemental Figure 5A, blue box). This contrasts with C57BL/6 mice where BCG vaccination had a minimal effect on CD4 T cell polyfunctionality. Significant decreases were observed for IFN-γ^+^TNF^+^ dual producers and IFN-γ single–positive cells in Mtb-infected BCG-vaccinated C57BL/6 mice, consistent with the observation that Th1 dominant response was associated with decreased bacterial burden in this background (Supplemental Figure 5A, purple box).

Finally, we sought to confirm that increased Th1/Th17 polyfunctionality was among the most significant correlates with BCG-mediated protection in CC strains with this phenotype. We performed a correlation analysis between the measured immune features and the normalized lung CFU from unvaccinated control and BCG-vaccinated mice. As a point of comparison, for C57BL/6 mice, IFN-γ–producing and polyfunctional CD4 T cells correlated with lung CFU. In CC037 mice, IFN-γ–producing CD8 T cells positively correlated with lung CFU. Importantly, many features were negatively correlated with lung bacterial burden in CC037 mice (Supplemental Figure 5B). The frequency of CD4^+^RORγt^+^ and antigen-stimulated IL-17 secretion and IFN-γ/IL-17 dual-producing CD4 T cells were significant even after correction for multiple testing, suggesting that these features were associated with protection following BCG. Finally, there was a strong correlation between the frequency of IFN-γ/IL-17 dual-producing CD4 T cells and lung CFU in CC037, CC072, CC001, and CC031 mice (Figure 9F).

## Discussion

We sought to determine how preexisting immunity to BCG modified the immune response to Mtb infection using a genetically diverse population of CC mice. An important outcome was our ranking of how BCG induced protection compared with control unvaccinated mice within each of these 24 CC strains, which expands the range of responses compared with the reference strain C57BL/6J. We kept constant the BCG strain, growth conditions, dose, and vaccination route. All mice were rested for 12 weeks after vaccination, challenged with the same Mtb strain, and analyzed 4 weeks after infection. The different CC strains were categorized as being either protected or unprotected by BCG against Mtb infection, based on whether vaccination led to a statistically significant reduction in lung bacillary burden measured 4 weeks after low dose aerosol infection. After controlling for these variables, the surprising result was that BCG protected only half of the CC strains tested. As these strains differed primarily in the genes and alleles they inherited from the CC founder strains, we concluded that the host genetic background had a major influence on whether BCG conferred protection against Mtb infection. These results extend our analysis of the 8 CC founder strains, which also vary in their ability to be protected by BCG vaccination (19). Not only do environmental mycobacteria and genetic variations in BCG strains used around the world interfere with the ability of BCG-induced protection, but we also suggest that host genetics should be considered a third important barrier to vaccine-mediated protection to TB.

Why doesn’t BCG induce protection in all CC strains? Protection was largely defined based on reductions in lung CFU 4 weeks after aerosol Mtb infection, which is a standard time point in murine vaccine studies. Interestingly, the CC strains that were not protected by BCG can be subdivided based on their lung pathology. In half of the unprotected CC strains (e.g., CC004), the BCG-vaccinated mice had changes in lung histology (e.g., more lymphocytic infiltrates) that differed from the unvaccinated controls. We concluded that, while immunization generated a memory recall response in these unprotected CC strains, it was insufficient to control lung CFU. Histological changes could not be identified in other unprotected strains (e.g., CC024). Indeed, we can find no evidence that CC024 mice generated an immune response to BCG. If the relative resistance of CC024 mice to Mtb infection is similar for other mycobacterial species, it is possible that BCG was eliminated quickly and failed to elicit a memory immune response.

Importantly, we wished to identify the components of the immune response stimulated by BCG, which were subsequently recalled after Mtb infection and associated with protection. We hypothesized that CC strains protected by BCG would share common immunological features. We postulated that comparing protected to unprotected CC strains would allow us to filter out components of the immune response that were elicited by vaccination or infection but not associated with protection. The T cell immune response following BCG vaccination and Mtb challenge was extensively characterized, as T cells are essential for protection against Mtb (26–30). Although considerable diversity was observed, BCG vaccination had little impact on the composition of T cells recruited and maintained in the lung after infection. Instead, the variability was in the numbers and types of T cells present in the lung during infection, which was largely shaped by the mouse genetic background. In contrast, functional skewing of the T cell cytokine production by BCG was evident in several of the protected CC strains. We developed a series of multivariate models to identify immune signatures associated with BCG-elicited protection against TB. These revealed that the correlates of protection common among CC strains were the decrease of several lung homogenate cytokines and increase in CD4 T cells expressing Foxp3 or RoRγT. When creating models specific to either resistant or susceptible CC mice that predicted protected versus unprotected mice, further correlates of protection emerged that could not be captured by the broader model. Importantly, there was substantial heterogeneity in the immune responses of protected mice, indicating multiple paths to protection.

Lung homogenate levels of IL-17 strongly correlated with lung CFU. However, the correlation between IL-17 and lung CFU was lost in BCG-protected CC strains after vaccination and challenge. Interestingly, lung IL-17 concentrations covaried with IL-1β and IL-6, 2 cytokines that are involved in skewing of Th17 cells, and with TNF, KC, MIP2, and G-CSF, all which are induced by IL-17 (Figure 3 and data not shown). IL-17 could be more generally important in driving lung inflammation than predicted by the C57BL/6 model. In contrast, the CD4 T cells from 4 of the 13 BCG-protected strains coproduced IFN-γ and IL-17 after Mtb infection. None of the 11 unprotected strains had this phenotype, and these dual producers were detected only in BCG-vaccinated mice and not in sham controls, nor were they detected in the C57BL/6 reference strain. Thus, IFN-γ/IL-17 dual-producing CD4 T cells are an example of an immune response primed by BCG but that is not manifested until the CD4 T cell response is recalled by Mtb infection. These cells are similar to Th1/17 cells, or Th1*, which are identified following TB infection and are associated with late-forming, low burden granulomas in a NHP TB infection model (31–33). However, an important difference is that IL-17 is generally not produced by Th1* cells after stimulation with Mtb antigens. Importantly, we found that dual IFN-γ/IL-17–producing CD4 T cells correlated with protection in several strains that were well-protected by BCG. Interestingly, within lung homogenates, IL-17 correlated with disease; conversely, IL-17-producing or RoRγt-expressing CD4 T cells correlated with protection. One possibility is that γΔ T cells or ILC3s, but not CD4 T cells, are the source of IL-17 in the lung homogenate. Alternatively, a cellular feature associated with Th1/17 cells other than IL-17 production, such the capacity for self-renewal, migration, or plasticity, is associated with Mtb control. These CC strains will be invaluable in further unraveling the role of IL-17 and IL-17-producing T cells in immunity to TB.

Our analysis is constrained by the data we collected, which focused on T cells and their function. However, the requirements for vaccine-mediated immunity to Mtb are not well defined and likely depend on the nature of the vaccine. A caveat of our analysis is that the analysis of certain immune components was cursory (e.g., B cells) or planned (e.g., the myeloid compartment and antibody responses). While we have not abandoned the search for universal correlates of vaccine-mediated protection that would be consistent across all CC strains, our data reveal several paths to protection. Although we controlled for as many variables as possible (e.g., BCG strain, dose, and route), another caveat is that other nonimmune factors may affect BCG efficacy. While BCG did not persist in the spleens of any CC strains, its persistence at other sites (e.g., draining LN) and the kinetics of its elimination could affect antimycobacterial immune responses. Recently, the gut microbiota was found to have a key role in modulating BCG in C57BL/6 mice (34). It is important to consider the interplay between host genetics and gut microbiota or persistence of whole-cell vaccines in vaccine efficacy.

While extrinsic factors that disrupt cell-mediated immunity increase the risk of developing TB, what drives the emergence of disease in most people is largely unknown. Host genetics affects susceptibility to TB both in mice and people, and genetic loci have been identified that increase TB risk (i.e., MSMD, polygenic) (20, 35–37). The factors that influence BCG efficacy remain controversial. We hypothesize that genetic factors contribute to variation in the effectiveness of BCG against pulmonary Mtb. Host polymorphisms affect BCG responses (reviewed in ref. 38). SNPs in innate and adaptive immune-related genes alter BCG induced responses, such as those in the TLR2, 6, 10, and TOLLIP genes, which affect BCG-induced cytokine secretion (39). Polymorphisms in genes associated with TB susceptibility, such as NRAMP1, IFN-γ and IL-10 also influence responses to BCG (40). Within the CC mice, we also observed variation in BCG-mediated cytokine production following TB infection. Our analysis showed that,while certain mouse genotypes could not be protected by BCG, the inherent susceptibility or resistance of the mouse strain was not a predictor of vaccine efficacy. This suggests that a priori, vaccines are appropriate interventions even in individuals that might have a genetic susceptibility to TB. As such, an in-depth analysis of the genetic basis of BCG efficacy in CC mice could identify biological pathways that are relevant to people.

One caveat is that we have only tested the efficacy of BCG in CC mice. The variation in host responses of CC mice could be leveraged as an ideal platform for testing the efficacy of preclinical vaccines in the context of a genetically diverse background. It is possible that vaccines that work through alternate mechanisms may be able to protect CC mice that are refractory to BCG vaccination. Instead of optimizing vaccine concepts in a single mouse genotype (e.g., C57BL/6 mice), identifying vaccines that are effective in numerous genotypes (i.e., genetically distinct individuals), as modeled by CC or diversity outbred (DO) mice, represents an alternative paradigm for preclinical vaccine development. Our data establish CC strains as what is, to our knowledge, a novel animal resource that can facilitate the discovery of correlates of vaccine-induced protection and accelerate preclinical vaccine testing.

## Methods

### Mice.

Mice were initially tested in 4 batches, with each batch including 6 different CC strains and C57BL/6J mice as a reference strain (Supplemental Table 1). Female C57BL/6/J (#0664) were purchased from The Jackson Laboratory. Female mice (6–10 weeks old) from 24 CC strains were purchased from the UNC Systems Genetics Core Facility (SGCF) between July 2018 and August 2019. The 24 CC strains used in this study include CC001/Unc, CC002/Unc, CC003/Unc, CC004/TauUnc, CC010/GeniUnc, CC011/Unc, CC012/GeniUnc, CC023/GeniUnc, CC024/GeniUnc, CC025/GeniUnc, CC026/GeniUnc, CC031/GeniUnc, CC032/GeniUnc, CC035/Unc, CC036/GemUnc, CC037/TauUnc, CC040/TauUnc, CC041/TauUnc, CC042/GeniUnc, CC044/Unc, CC045/GeniUnc, CC059/TauUnc, CC061/GeniUnc, and CC072/TauUnc. More information regarding the CC strains can be found at http://csbio.unc.edu/CCstatus/index.py?run=AvailableLines.information Male mice were excluded because their aggressive behavior made it impractical to cohouse them for the duration of these experiments.

### Bacterial strains.

Master stocks of H37Rv expressing yellow fluorescent protein (sfYFP) (17) and BCG (SSI strain) were grown and a large batch of aliquots were frozen. All BCG vaccinations and Mtb challenges used the same batch of bacteria. BCG (10^6^ CFU/mL 0.04% Tween/PBS) was administered subcutaneously (100 μL/injection),

### Aerosolized Mtb infection of mice.

Mice were infected by the aerosol route as previously described (21). Frozen bacterial stocks were thawed and sonicated for 1 minute, then diluted into 5 mL of 0.01% Tween-80 (MilliporeSigma) in PBS (Gibco). The diluted bacterial suspension was aerosolized using a Glas-Col chamber (Terre Haute). The average number of bacteria delivered into the lung was determined for each experiment by plating lung homogenate from 4–5 mice 24 hours after infection and ranged between 40–150 CFU/mouse.

### Bacterial burden in lung and spleen.

Infected mice were euthanized at predetermined time points, and the left lung or whole spleen were homogenized using 2 mm zirconium oxide beads (Next Advance) in a FastPrep homogenizer (MP Biomedicals). Tissue homogenates were serially diluted and plated on 7H11 agar plates (Hardy Diagnosis). CFU was enumerated after 19–21 days of incubation at 37°C and 5% CO_2_.

### Lung cell preparation.

Single cell suspensions were prepared by homogenizing right lungs using a GentleMACS tissue dissociator (Miltenyi Biotec), digesting with 300 U/mL collagenase (Sigma-Aldrich) in complete RPMI (10% FBS [Sigma-Aldrich], 2 mM L-Glutamine, 100 units/mL Penicillin/Streptomycin, 1 mM Na-Pyruvate, 1× nonessential amino acids, 0.5× minimal essential amino acids, 25 mM of HEPES [all from Gibco], and 7.5 mM of NaOH [Sigma-Aldrich]) at 37°C for 30 minutes and followed by a second run of dissociation using the GentleMACS. Suspensions were then sequentially filtered through a 70 μm strainer, treated with ACK lysis buffer (Gibco), then filtered through 40 μm strainers.

### Flow cytometric analysis.

Cells were first stained with Zombie Fixable Viability dye (BioLegend) for 10 minutes at room temperature (RT), after which cells were stained with antibodies in autoMACS running buffer (Miltenyi) containing 5 μg/mL of anti-mouse CD16/32 (BioXcell) for 10 minutes at 4°C. The antibodies used in this study are listed in Supplemental Table 3. In some experiments, MAIT cells were identified using the MR-1-PE tetramer (NIH tetramer core facility) at 37°C for 1 hour. Cells were then stained with surface antibody cocktail for 20 minutes at 4°C. To measure transcription factor (TF) expression, cells were fixed and permeabilized for 30 minutes at RT, followed by staining for 30 minutes with antibodies to the different TF at RT using the Foxp3/TF staining buffer set (Thermo Fisher Scientific). Samples were acquired on the Aurora (Cytek).

For intracellular staining, cells were first stimulated for 5 hours with either anti-CD3 antibodies (1 μg/mL, BioLegend) or MTB300 peptide megapool (24) which contains 300 peptides representing 90 antigens, and contains epitopes recognized by both murine CD4 and CD8 T cells. GolgiPlug (BD Biosciences) was also given at this time (0.5 μg/well). Viability and surface staining were performed as described, after which cells were fixed instead with Fixation/Permeabilization kit for 20 minutes at 4°C. Cells were then stained with an intracellular cytokine antibody cocktail for 20 minutes at 4°C.

To inactivate the bacteria, samples were fixed with 1% paraformaldehyde (Thermo Fisher Scientific) in PBS for 1 hour at room temperature and then washed with MACS buffer. For both cell surface and intracellular markers, new markers were introduced in the repeat experiments to broaden the immune phenotyping. Flow data were analyzed using FlowJo v10.7.1. Polyfunctionality was examined using the SPICE platform (41).

### LEGENDplex cytokine analysis.

Lung cells were stimulated with MTB300 peptide megapool (1 μg/mL) or anti-CD3 (1 μg/mL) at a concentration of 10^6^ cells/mL for 24 hours. Culture supernatants were filtered through 0.2 μm filter, after which cytokine levels were measured with LEGENDplex kits from BioLegend. Specifically, we used their 13-plex mouse T helper cytokine panel (IFN-γ, TNF, IL-2, IL-4, IL-5, IL-6, IL-9, IL-10, IL-13, IL-17a, IL-17f, IL-21, and IL-22) as well as their 13-plex mouse proinflammatory chemokine panel (RANTES, MIP-3α, Eotaxin, TARC, KC, MCP-1, MIG, IP-10, MIP-1α, MIP-1β, BLC, LIX, and MDC) for a total of 26 cytokines and chemokines.

### Lung homogenate.

Cytokines and chemokines in lung homogenates obtained 4 weeks after infection were measured using the 31-plex discovery Assay (MD31) from Eve technologies.

### Histology analysis.

The right middle lung lobe from each mouse was immersion fixed in 10% formalin (Anatech) immediately following euthanasia. Lung tissue was processed, embedded in paraffin, sectioned at 5 μm, and stained with H&E at the Morphology Core Facility at UMass Chan Medical School (Worcester, Massachusetts, USA) for histological examination by a board-certified veterinary pathologist. Blind classification (BCG versus unvaccinated) within each CC strain was determined based on the assumption that BCG vaccination would have 3 possible effects on lungs: (a) reduce necrosis/neutrophilic infiltrates; (b) increase lymphocytes and/or plasma cell abundance; and (c) reduce granuloma size. Five of the 24 CC strains were not evaluated because of logistical issues. Accuracy of the blind classification was assessed by an independent investigator. All glass slides were subsequently reevaluated to examine and describe histopathology features attributable to BCG vaccination and attempt to find unique responses more closely. Glass slides were then digitized by Vanderbilt University Medical Center (Nashville, Tennessee, USA) and whole images generated using Aperio ScanScope at 400 times normal magnification with jpeg compression. Representative images were captured using Aperio ImageScope magnified 40 and 400 times and imported into GraphPad Prism 9 to generate the panel Figure. Digital images were not adjusted. Whole slide imaging and quantification of immunostaining were performed in the Digital Histology Shared Resource (DHSR) at Vanderbilt University Medical Center.

### Univariate correlations.

To evaluate how C57BL/6 mice, protected CC strains, and nonprotected CC strains compared in their relationships between lung cytokine production and Mtb burden, a series of univariate Pearson correlations were conducted. The Pearson correlation between the natural log (ln) of lung homogenate cytokine and chemokine concentrations and the log_10_ lung CFU or spleen CFU was determined for C57BL/6 mice, protected CC strains, and nonprotected CC strains. A Benjamini-Hochberg multiple hypothesis correction was applied to account for the number of correlations calculated. A further comparison of the Pearson correlation coefficients between the BCG-vaccinated and unvaccinated mice within the C57BL/6, protected CC, and nonprotected CC categories was conducted. To do so, the correlation coefficients underwent a Fisher’s z transformation and then were compared by a z test, with the reported *P* values (“pdiff”) being Benjamini-Hochberg corrected *P* values of this comparison.

### Multivariate analyses.

Multivariate analyses involved combining all 4 immunological data sets in this study: the lung homogenate cytokine/chemokine measurements, the ICS, the LEGENDplex cytokine analysis, and the T cell phenotypic flow cytometry. The analyses also included all CC mice used in the study in addition to the control C57/BL6 mice.

For the ICS, the data were analyzed as combinatorics of the 7 markers measured in order to best capture polyfunctionality and further divided by CD4 T cell, CD8 T cell, CD4^–^CD8^–^ T cell, and CD3^–^ categories. The features were filtered by a minimum mean and variance percentage of 10^–6^. Only MTB300-stimulated samples were used from the ICS data set. The flow cytometry data was in the form of percentages of CD3^+^ or CD3^–^ cells, while the immunoassay data was in the form of concentrations. The combined data set was ln transformed and subsequently z scored by measurement across all samples. For models that involved BCG-induced protection, these z-scored values were further transformed by subtracting the features from each vaccinated mouse and the mean of the features of the unvaccinated mice from the same mouse strain.

For the PLSR and PLSDA models, elastic net feature selection was performed in 100 trials of 5-fold cross validation, where features that were present in at least 80% of trials were included in the final model. Each model was compared with 2 null models: 1 with permuted lung CFUs and 1 with randomly chosen features. The mean squared error of the prediction of lung CFU (for the PLSR) or the classification accuracy (for the PLSDAs) was compared with that of the null models in 50 trials of 5-fold cross validation.

### Statistics.

Statistical analysis was performed using Graphpad Prism 9. *P* values were calculated using unpaired *t* test, 1-way ANOVA, or 2-way ANOVA, as indicated in the figure legends. Multivariate analyses were performed in R (v3.6.0 and 4.0.4) using the ropls Bioconductor package.

### Study approval.

Studies were conducted using the relevant guidelines and regulations and were approved by the IACUC at the University of Massachusetts Medical School (UMMS) (Animal Welfare A3306-01), using the recommendations from the Guide for the care and Use of Laboratory Animals of the National Institutes of Health and the Office of Laboratory Animal Welfare.

## Author contributions

RL and SMB were responsible for conceptualization and methodology. RL, DNG, TW, AFO, and KC conducted the investigation. RL, DNG, and GLB performed the formal analysis. RL, DNG, SA, and SMB wrote the original draft of the manuscript. RL, DNG, CSLA, GLB, MTF, CMS, and SMB reviewed and edited the manuscript. RL, DAL, and SMB supervised the project. SMB acquired funding for the project.

## Supplementary Material

Supplemental data

## Version 1 05/18/2023

In-Press Preview

## Version 2 07/03/2023

Electronic publication

## Version 3 01/29/2024

Acknowledgments section updated.
