# Supplementary material for: Host genetic background is a barrier to broadly effective vaccine–mediated protection against tuberculosis
Source: J Clin Invest. 2023 Jul 3;133(13):e167762. doi: 10.1172/JCI167762 (PMC10313364; doi:10.1172/JCI167762)
Supplement: Supplemental data [file jci-133-167762-s010.pdf]

Table S1. A description of the CC strains analyzed in this study						
**CC strain**	**MHC Haplotype[a]**	**Batch[b]**	**ΔLung CFU[c]**	**ΔLung CFU[d]**	**ΔSpleen CFU[e]**	**ΔSpleen CFU[f]**
CC042/GeniUnc	b (129)	1	1.65	0.96	1.00	1.32
CC025/GeniUnc	pwk	3	1.40	n/a	0.43	n/a
CC059/TauUnc	b (129)/a	2	1.39	n/a	1.17	n/a
CC037/TauUnc	b (B6)	1	1.37	1.84	1.07	1.05
CC031/GeniUnc	b (B6)	3	1.22	1.35	1.26	1.60
CC023/GeniUnc	b (129)	1	1.17	-0.09	1.47	0.32
CC032/GeniUnc	b (B6)	1	1.16	n/a	1.06	n/a
CC011/Unc	g7	3	1.04	n/a	2.08	n/a
CC072/TauUnc	b (B6)	2	0.97	1.37	1.06	1.41
CC001/Unc	b (B6)	4	0.94	1.45	2.79	1.45
CC026/GeniUnc	cast	2	0.93	1.05	3.75	1.87
CC061/GeniUnc	b (129)	1	0.93	n/a	1.58	n/a
CC035/Unc	wsb	4	0.91	n/a	1.13	n/a
CC002/Unc	b (129)	3	0.83	n/a	0.46	n/a
CC012/GeniUnc	wsb	4	0.79	n/a	0.89	n/a
CC036/Unc	g7	3	0.62	n/a	1.84	n/a
CC010/GeniUnc	b (B6)	1	0.53	n/a	0.92	n/a
CC045/GeniUnc	z	4	0.24	n/a	0.13	n/a
CC024/GeniUnc	cast	2	0.15	0.24	0.38	0.62
CC041/TauUnc	b (129)	2	0.14	0.94	0.71	0.94
CC044/Unc	b (B6)/g7	4	0.08	0.13	0.12	0.39
CC003/Unc	g7	3	0.07	-0.61	0.77	0.15
CC004/TauUnc	b (129)	4	0.05	0.08	-0.16	-0.12
CC040/TauUnc	b (B6)	2	-0.64	-0.28	-0.35	1.14

a) Indicates MHC haplotype of each CC strain

b) Indicates batch that the CC strain were part of for the initial round of immune characterization

c, e) Reduction of lung and spleen CFU of the initial round of CC infection (Δlog10[CFU])

d, f) Reduction of lung and spleen CFU of the repeat cohort (Δlog10[CFU])

Table S1. Protection of CC strains.

The formal name and H-2 haplotype of the 24 CC strains analyzed including the original batch. The reduction in Mtb burden attributed to BCG vaccination in the lung and spleen (ΔLung and ΔSpleen CFU), are indicated. Thirteen CC strains were selected to confirm the initial findings. Red text indicates experiments where BCG enhanced Mtb burden. Highlighted values indicate discordance between the initial experiment and the repeat experiment. C57BL/6 mice were included in all experiments as a control.

Table S2. Summary of histological findings			
[a] Strain	[b] BCG-induced protection	[c] Change in pathology	[d] How did the vaccinated mice differ?
CC003	N	N	No differences. Both groups had mild lesions with perivascular lymphocytic cuffs and a few macrophage-rich lesions
CC041	N	N	No differences. Both groups had mild lesions with perivascular lymphocytic cuffs and a few macrophage-rich lesions
CC024	N	N	No differences. Both groups had small hard-to-find lymphocytic lesions with perivascular and peribroncial lymphocytic cuffs
CC002	N	Y	Ctrl had mild lesions with perivascular lymphocytic cuffs. BCG mice had larger moderate sized macrophage-rich lesions. Lymphocytes were similarly abundant.
CC004	N	Y	Ctrl mice had mild to moderate lesions with lymphohistiocytic infiltrates. BCG mice have more lymphocytes with regions of dense perivascular/peribronchiolar lymphocytic cuffs.
CC036	N	Y	Ctrl mice have mild disease with lymphohistiocytic infiltrates. BCG mice also have lymphohistiocytic infiltrates with few very dense lymphocytic areas
CC040	N	Y	Ctrl mice have moderate necrotizing lesions with few lymphocytes. BCG mice have mild disease - small & hard to find lesions that are lymphocyte rich without necrosis.
CC044	N	Y	Unusual. Ctrl mice have lymphohistiocytic infiltrates with dense lymphocytic cuffs. BCG mice have lymphohistiocytic infiltrates with dense lymphocytic cuffs and lymphoid aggregates/follicules in parenchyma.
CC061	P	N	No differences. Unusual. Mild lesions. Both groups have dense lymphoid aggregates/follicles in parenchyma. Also, perivascular lymphocytic cuffs.
CC072	P	N	No differences. Mild disease. perivascular lymphocytic cuffs with a few macrophage-rich lesions. One mouse has a foreign-body granulomatous bronchitis/pneumonia with intralesional food/bedding/plant.
C57BL/6	P	Y	Ctrl mice had mild to moderate lymphohistiocytic lesions. BCG mice had with more, dense lymphocytic infiltrates
CC001	P	Y	Ctrl mice had moderate lesions with lymphohistiocytic infiltrates and equivocal necrosis. BCG mice had mild lesions with perivascular lymphocytic cuffs and a few macrophage-rich lesions without necrosis
CC023	P	Y	Ctrl mice had moderate to severe lesions with necrosis and minimal lymphocytes. BCG mice have moderate lesions with more lymphocytes and less necrosis.
CC025	P	Y	Ctrl mice have moderate disease with mild lymphocytic infiltrates and necrosis. BCG mice have abundant dense perivascular/peribronchiolar lymphocytic ifiltrates and lymphohistiocytic granulomas and less necrosis.
CC026	P	Y	Ctrl mice had moderate lesions with lymphohistiocytic infiltrates. BCG mice had mild lesions with lymphohistiocytic infiltrates and a few very dense lymphocytic areas
CC031	P	Y	Ctrl mice have moderate disease with lymphohistiocytic infiltrates. BCG mice have abundant dense perivascular/peribronchiolar lymphocytes and lymphohistiocytic granulomas
CC032	Protected	Y	Ctrl mice have mild disease with lymphohistiocytic infiltrates. BCG mice have more lymphocyte rich lesions that are small & hard to find
CC037	Protected	Y	Ctrl mice have moderate disease with lymphohistiocytic infiltrates. BCG mice have abundant dense perivascular/peribronchiolar lymphocytes and lymphohistiocytic granulomas
CC042	Protected	Y	Ctrl mice have mild to moderate disease with few lymphocytes and necrosis. BCG mice have mild disease with more lymphocytes, less necrosis
CC059	Protected	Y	Ctrl mice had mild disease with lymphohistiocytic infiltrates with small regions of necrosis. BCG mice have abundant dense perivascular/peribronchiolar lymphocytes and lymphohistiocytic granulomas without necrosis

Table S2. Pathological descriptions of changes lung histology attributed to BCG vaccination in Mtb-infected CC mice. A summary of the pathological features in the CC strains analyzed. A) Nineteen of the 24 CC strains were analyzed and the ability of BCG to protect each strain (see Figure 1) is indicated as N (not protected) or P (protected). Histological sections were compared between unvaccinated and BCG vaccinated mice following Mtb infection. A change in pathology attributed to BCG is indicated as Y (yes) or N (no). A description of the histological changes between control and BCG vaccinated mice is provided.

Table S3: Flow cytometry reagents

Specificity	Clone	Fluor	Source	Catalog #
Foxp3	FJK-16S	Alexa488	Invitrogen	53-5773-82
Gata3	TWAJ	PerCp	Invitrogen	46-9966-62
Tbet	4B10	PacificBlue	Biolegend	644808
RoRγt	Q31-378	BV650	BD	564722
CD25	PC61	BV785	Biolegend	102051
CD11a	M17/4	BV711	Biolegend	740676
CD44	IM7	PE	Biolegend	103030
PD1	29F.1A12	BV605	Biolegend	135220
CD3	145-2CL1	PerCP	Biolegend	100328
CD4	GK1.5	Alexa700	Biolegend	100430
CD8a	53-6.7	APC	Biolegend	100766
CD62L	MEL-14	BV570	Biolegend	104433
CD127	A7R34	PE	Biolegend	135032
CD103	2E7	BV421	Biolegend	121422
IL4	11B11	Alexa488	BD	557728
TNF	MP6-XT22	BV421	Biolegend	506328
IL2	JES6-5H4	PE-Cy7	Biolegend	504832
IL17	TCH11-18H10.1	BV650	Biolegend	506930
IL10	JES5-16E3	BV605	Biolegend	505031
IFNg	XMG1.2	APC	Biolegend	505810
CD107a	LAMP-1	PE	Biolegend	121612
CD19	6D5	PE-Cy5	Biolegend	115510
MR1	(tetramer)	PE	NIH tetramer core	
MR1	(tetramer)	PE	NIH tetramer core	
N/A	N/A	Aqua	Biolegend	423102

Table S3. Antibodies used in this study.

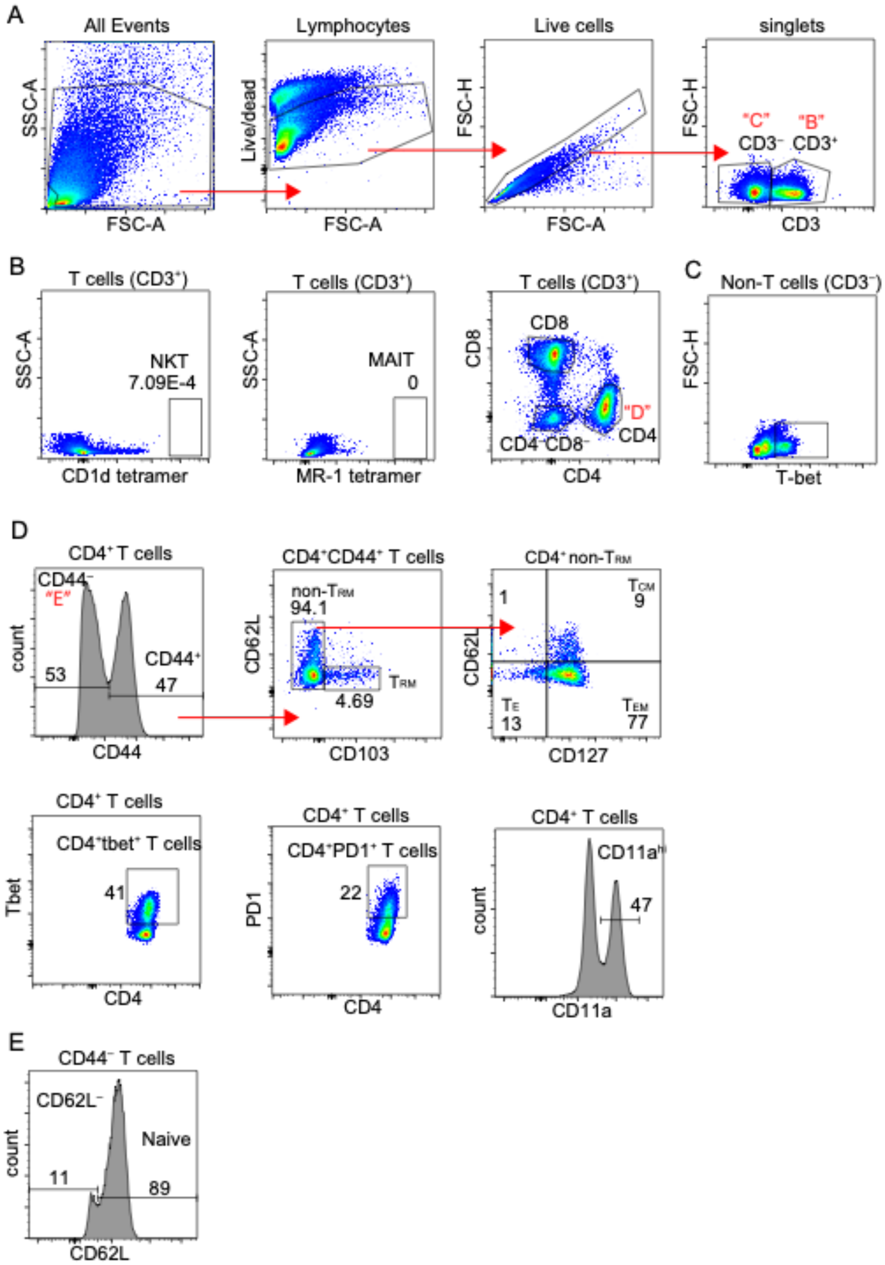

Figure S1. Gating strategy for analysis of T cell phenotypes.

(A) Lymphocytes were identified using FSC and SSC properties, and viability dyes were used to exclude dead cells. Singlet gates excluded doublets and T cells and non-T cells were distinguished using the lineage marker CD3. (B) NKT and MAIT cells were identified from the $CD3^+$ gate using antigen-loaded CD1d and MR1 tetramers, respectively. $CD4^+$, $CD8^+$ and $CD4^-CD8^-$ T cells were identified in the T cell population. (C) Markers, such as T-bet expression, were used to characterize the $CD3^-$ population. (D) CD44 identified antigen-experienced CD4 T cells. CD103 identified resident memory T cells (T_{RM}). From the $CD103^-$ fraction, CD127 and CD62L expression identified central memory (T_{CM}), effector (T_E), and effector memory (T_{EM}). Subset identification of various T cell subsets (ex: $Tbet^+CD4^+$ T cells) and activation markers (ex: PD1, CD11a) were assessed among $CD4^+$, $CD8^+$ and $CD4^-CD8^-$ T cell subsets. (E) Naïve T cells were identified within the $CD44^-$ compartment using CD62L expression.

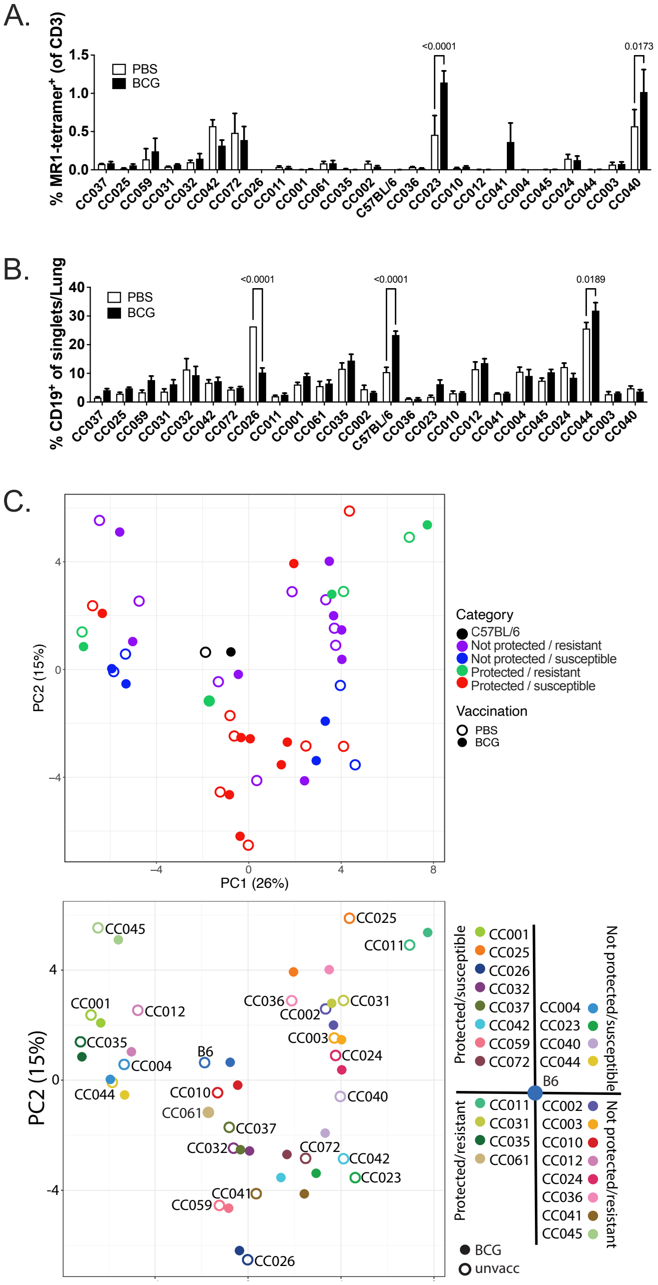

Figure S2. Frequencies of non-conventional T cells in CC mice. Frequencies of (A) MR-1-restricted MAIT cells and (B) CD19$^+$ B cells were measured in the lungs of unvaccinated or BCG vaccinated CC mice at 4 weeks post Mtb infection. The data in the graphs represent the mean ± SEM of one experiment. Two-way analysis of variance with the Benjamini and Hochberg multiple comparison method. The FDR was set to 0.05 and the numbers in the figures are the q value. (C) PCA of the T cell phenotyping data. Top: Each point represents the average of 5 mice within a given mouse strain that are either BCG vaccinated (closed symbols) or unvaccinated (open symbols). Each color represents a different quadrant from Figure 1C. Bottom: The PCA from Figure 4F is reproduced here for side-by-side comparison. Each color represents a different mouse strain, and the unvaccinated group (open symbols) is labelled with the CC strain name.

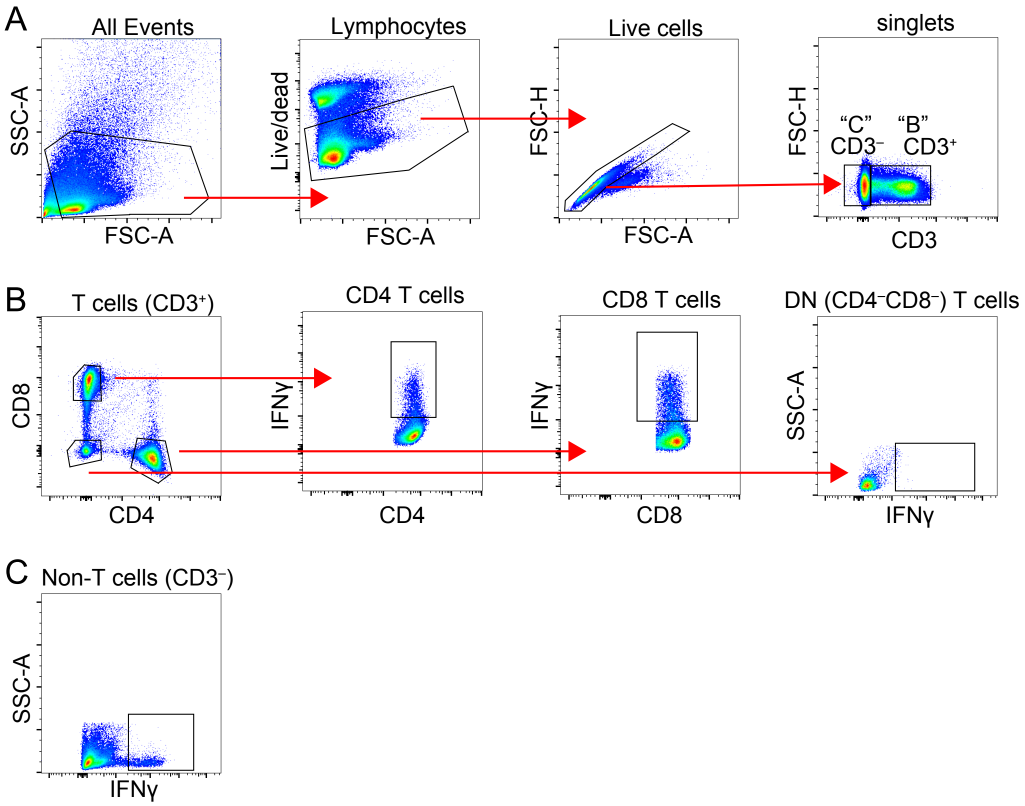

Figure S3. Gating strategy for analysis of intracellular cytokine production by T cells.

(A) Lymphocytes were identified by their FSC and SSC properties, after which viability dyes were used to exclude dead cells. Singlet gates were used to exclude doublets, after which T cells were identified using the lineage marker CD3. (B) CD4, CD8 and $CD4^{-}CD8^{-}$ T cells were identified in the $CD3^{+}$ population. Cytokine expression was determined in all 3 T cell sub-populations. (C) Cytokine expression was also examined within the $CD3^{-}$ population.

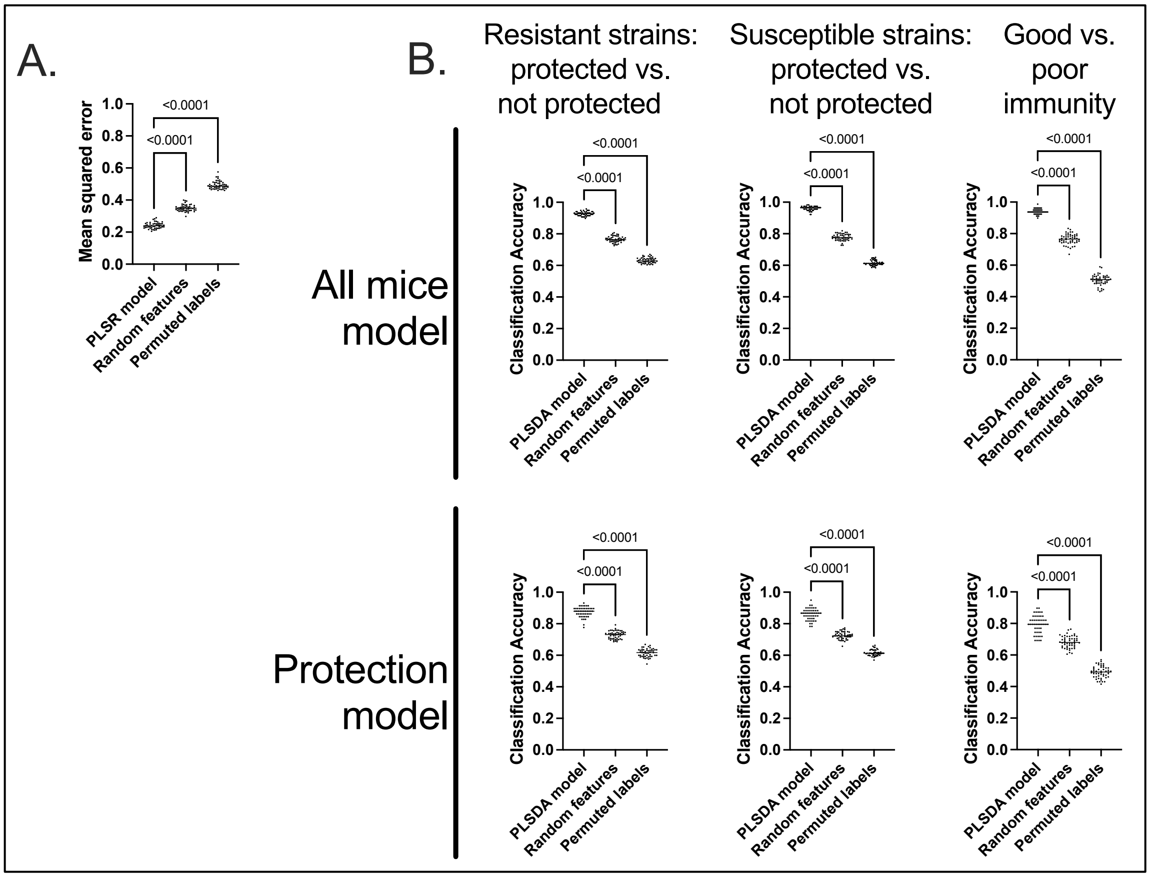

Figure S4. Multivariate model accuracies.

(A) The accuracy of our protection model (Figure 6) was compared to that of two different types of null models, where our measure of accuracy was mean squared error of prediction of Δlog (lung CFU). Our PLSR model performed significantly better than both a null model consisting of random features instead of our elastic-net chosen features and a null model where the outcomes of Δlog (lung CFU) were scrambled. Our PLSR model and each of the null models were run 50 times, and each dot represents one of these trials. Labeled p-values are from one-way ANOVAs.

(B) Our models highlighting the immune features that distinguish pairs of protection/susceptibility categories (Figure 7) were compared to two null models as in (A). Each of our models, both the ones involving all mice and the protection models were significantly better than both null models. Here, the accuracy metric is classification accuracy. Labeled p-values are from one-way ANOVAs.

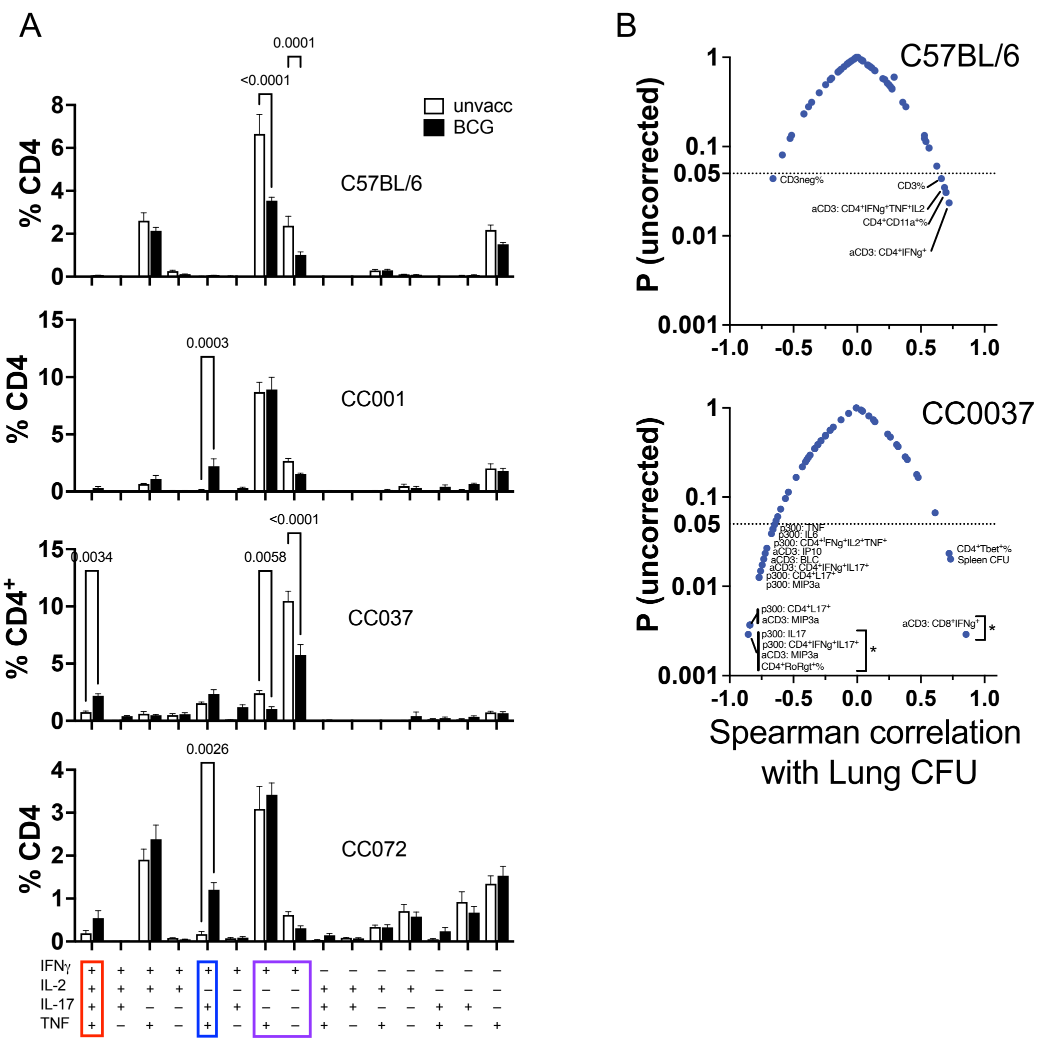

Figure S5. Immunity in select CC strains.

(A) Boolean gating was used to identify sub-populations among CD4 T cells following MTB300 megapool stimulation. An increase in IFNγ$^+$IL-17$^+$ CD4 T cells (that also produced TNF or IL-2) were detected in CC037, CC072, and CC001 but not in C57BL/6. In contrast, a noticeable decrease in the frequency of IFNγ "single positive" and IFNγ$^+$TNF$^+$ CD4 T cells was observed in C57BL/6 and CC037 mice. The data in the graphs represent the mean ± SD of two experiment. One-way analysis of variance with corrected with the Benjamini and Hochberg multiple comparison method (FDR = 0.05). q values are shown. (B) Volcano plots. Correlation between 60 immune features and the lung CFU determined for CC037 and C57BL/6 mice (from Batch 1). Dotted line, p = 0.05. *, q < 0.05, corrected with the Benjamini and Hochberg multiple comparison method (FDR = 0.05).
